# Sequence stratigraphy of the syn-rift miocene succession in the Abu Rudeis-Sidri Field, Gulf of Suez, Egypt

**DOI:** 10.1038/s41598-025-26923-z

**Published:** 2025-11-29

**Authors:** Ehab M. Assal, Sherif Farouk, Mohamed A. Omran, Nancy Belal, Mohammad A. Sarhan

**Affiliations:** 1 Geology Department, Faculty of Science, Damietta University, New Damietta City, Damietta, 34517 Egypt; 2 Exploration Department, Egyptian Petroleum Research Institute (EPRI), 1 Ahmed El-Zomor Street, Nasr City, Cairo, 11727 Egypt

**Keywords:** Biostratigraphy, Sequence stratigraphy, Wireline logs, Abu Rudeis–Sidri Field, Miocene, Gulf of Suez, Climate sciences, Solid Earth sciences

## Abstract

The syn-rift Miocene succession of the Gulf of Suez remains poorly constrained, with persistent uncertainties in rift initiation timing, paleoenvironmental reconstruction, and the interplay between tectonics and eustasy—factors that complicate stratigraphic correlation and hydrocarbon exploration. This study aims to refine the chronostratigraphic framework, reconstruct depositional environments, and develop a detailed sequence stratigraphic model for the Abu Rudeis–Sidri Field in the east-central Gulf of Suez through integration of high-resolution foraminiferal biostratigraphy, wireline logs, and seismic data from four wells (ARM-7, ARS-6, SIDRI-20, SIDRI-9). Planktonic and benthic foraminifera constrain the succession to the early Burdigalian–early Langhian, encompassing the *Globigerinoides altiaperturus–Catapsydrax dissimilis*, *Trilobatus trilobus*, and *Praeorbulina glomerosa* zones. Ten benthic biofacies define paleodepths from inner shelf (< 50 m) to upper slope (> 150 m), enabling reconstruction of depositional environments and relative sea-level trends. Four third-order depositional sequences (SQ1–SQ4) are recognized, bounded by regionally mappable sequence boundaries and maximum flooding surfaces. Stacking patterns reveal deepening from SQ1 to SQ3 followed by shoaling and lagoonal restriction in SQ4. Tectonic subsidence and block tilting exerted primary control on accommodation, whereas eustatic influence is expressed mainly at flooding surfaces. Correlation with regional and global sea-level curves shows partial alignment with Burdigalian–Langhian cycles, underscoring the dominance of local tectonics. This refined framework enhances understanding of syn-rift sedimentation and provides predictive insights for hydrocarbon exploration in rift-related basins.

**Supplementary Information:**

The online version contains supplementary material available at 10.1038/s41598-025-26923-z.

## Introduction

**Fig. 1:**
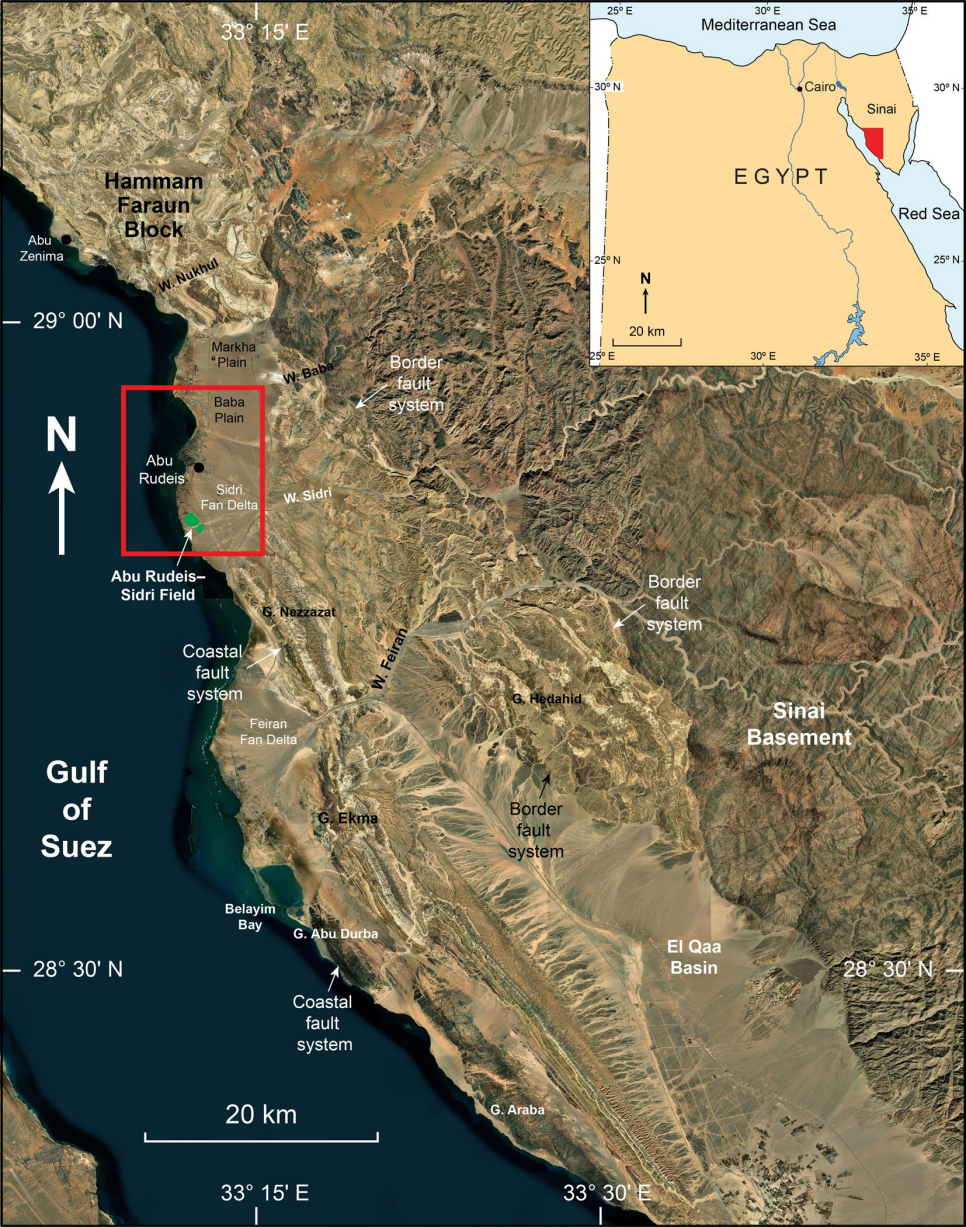
High-resolution world imagery base map (accessed through Global Mapper Pro v26.2) illustrating the eastern margin of the Gulf of Suez Basin. The red rectangle marks the location of the Abu Rudeis-Sidri Field.

The Gulf of Suez is a northeast-trending continental rift that constitutes the northern termination of the Red Sea rift system (Fig. 1). Its geological setting includes a variety of reservoir types ranging from clastic to carbonate sequences, which have been extensively studied to better understand their petrophysical and structural characteristics^1–3^. Extension began in the Late Oligocene–Early Miocene as the Arabian plate moved away from the African plate, creating a system of half-grabens bounded by NW-oriented normal faults^4–8^.

Over the past five decades, numerous studies have addressed the basin’s geometry, tectono-sedimentary evolution, and the development of syn-rift depocenters^4,9–21^. This rift now constitutes a mature petroleum province, where syn-rift Miocene strata provide the principal reservoirs, and pre-rift Cretaceous–Paleogene units form important secondary targets^22^. Structural traps largely reflect the complex tectonic history that accompanied rifting.

National schemes proposed by the Egyptian General Petroleum Corporation^23^and the National Committee of Geological Sciences^24^were later refined at field scale, yet the pronounced lateral lithologic variability still yields conflicting local subdivisions (^25–28^, and references therein). The syn-rift succession is lithologically diverse, comprising shales, sandstones, carbonates, and evaporites, reflecting a range of marine depositional environments influenced by local tectonics and relative sea-level fluctuations^12,14^. These facies transitions reflect local subsidence patterns, tectonic segmentation, and sea-level fluctuations. Regional correlation across adjacent fault blocks in the central Gulf of Suez is challenging, as rapid lateral facies changes and differential subsidence complicate consistent stratigraphic matching.

Biostratigraphic zonations based on benthic and planktonic foraminifera (e.g^29–31^.,) and calcareous nannofossils^32,33^have improved age control, yet coarse-grained and marginal-marine intervals remain difficult to date owing to sparse fossils and low organic content. Consequently, correlations often rely on wireline-log motifs, introducing appreciable uncertainty.

At local to semi-regional scales, sequence-stratigraphic frameworks have been developed from seismic reflection data, well logs, and outcrop analysis^34–38^.

Most models recognize multiple third-order sequences bounded by regional unconformities, commonly interpreted as tectonically driven lowstands. However, the internal architecture of these sequences, including their stacking patterns, systems tracts, and lateral continuity, remains poorly constrained, especially offshore. Integrating offshore subsurface data with onshore sections has begun to reveal how tectonics and eustasy controlled facies distribution and reservoir quality^28,39–41^.

The Abu Rudeis–Sidri Field represents a structurally complex sector in the central Gulf of Suez, which remains less well documented than the northern and southern rift provinces. In contrast to regions with extensive outcrop control, the Markha Plain is largely devoid of significant surface exposures, making well and seismic data essential for reconstructing its tectono-stratigraphic evolution. The field has been under production since the 1950 s, primarily from Miocene sandstone reservoirs (Nukhul, Rudeis, and Kareem formations), with more than 60 wells drilled to date. Accordingly, this study addresses a key ‘missing piece’ in the rift-basin architecture, the implications of which extend beyond hydrocarbon exploration to the development of refined, generic rift models and syn-rift reservoir analogues worldwide.

Despite abundant data, the precise timing of rift initiation remains uncertain—particularly across the Oligocene–Miocene transition and the age of the Nukhul Formation, which remains loosely constrained between the Chattian and Aquitanian. Fine-scale resolution of syn-rift paleo-environments, their reservoir characteristics, and the interplay between tectonics and sea-level change are likewise insufficiently constrained. This study seeks to address these challenges by: (1) developing a high-resolution biostratigraphic scheme for the Miocene subsurface using foraminiferal assemblages to delineate unconformities and refine ages; (2) reconstructing depositional environments by integrating benthic foraminiferal data with lithofacies interpreted from wireline logs; (3) proposing an updated depositional model for the syn-rift succession; and (4) constructing a sequence stratigraphic framework by integrating biostratigraphy, wireline logs, and seismic data, although the resolution was insufficient to delineate systems tracts or detailed stacking patterns. The findings will support hydrocarbon exploration in analogous rift basins and contribute to a broader understanding of syn-rift tectono-sedimentary processes.

## Geological background

### Structural setting

The Gulf of Suez is a northwest-trending, failed intracontinental rift basin that formed during the Late Oligocene to Early Miocene due to the north-eastward separation of the Arabian plate from the African plate^1,2,5–8^. Extension was initially orthogonal to the rift axis (N65°E) but changed during the Middle Miocene to Quaternary to a more oblique NNE direction in response to sinistral strike-slip motion along the Gulf of Aqaba–Dead Sea transform system^42,43^. The rift architecture consists of three main dip provinces; northern, central, and southern, each composed of northwesterly trending half-grabens bounded by major listric normal faults, which exhibit a change in polarity along the axial length of the rift^42,44^. These depocenters are separated by accommodation zones that localize structural complexity and facies transitions (^45–47^). Rift initiation in the Gulf of Suez appears to have been diachronous, with evidence indicating a south-to-north propagation of extensional activity. This diachroneity complicates basin-wide correlations and underscores the importance of precise age constraints within the central dip province.

This study focuses on the Markha Plain in the central dip province, a southwest-dipping fault block bounded by the East–West trending, south dipping Baba–Markha Fault Zone to the north, and the Nezzazat Coastal Fault and North Baba Fault Zone to the southeast^48,49^. The block forms part of a half-graben system, where tilted fault blocks and syn-rift stratigraphy are controlled by the activity of the bordering coastal faults. Structural cross sections reveal northeast-dipping strata against southwest-dipping normal faults that accommodated Miocene subsidence and syn-rift sedimentation.

### Stratigraphic framework

**Fig. 2:**
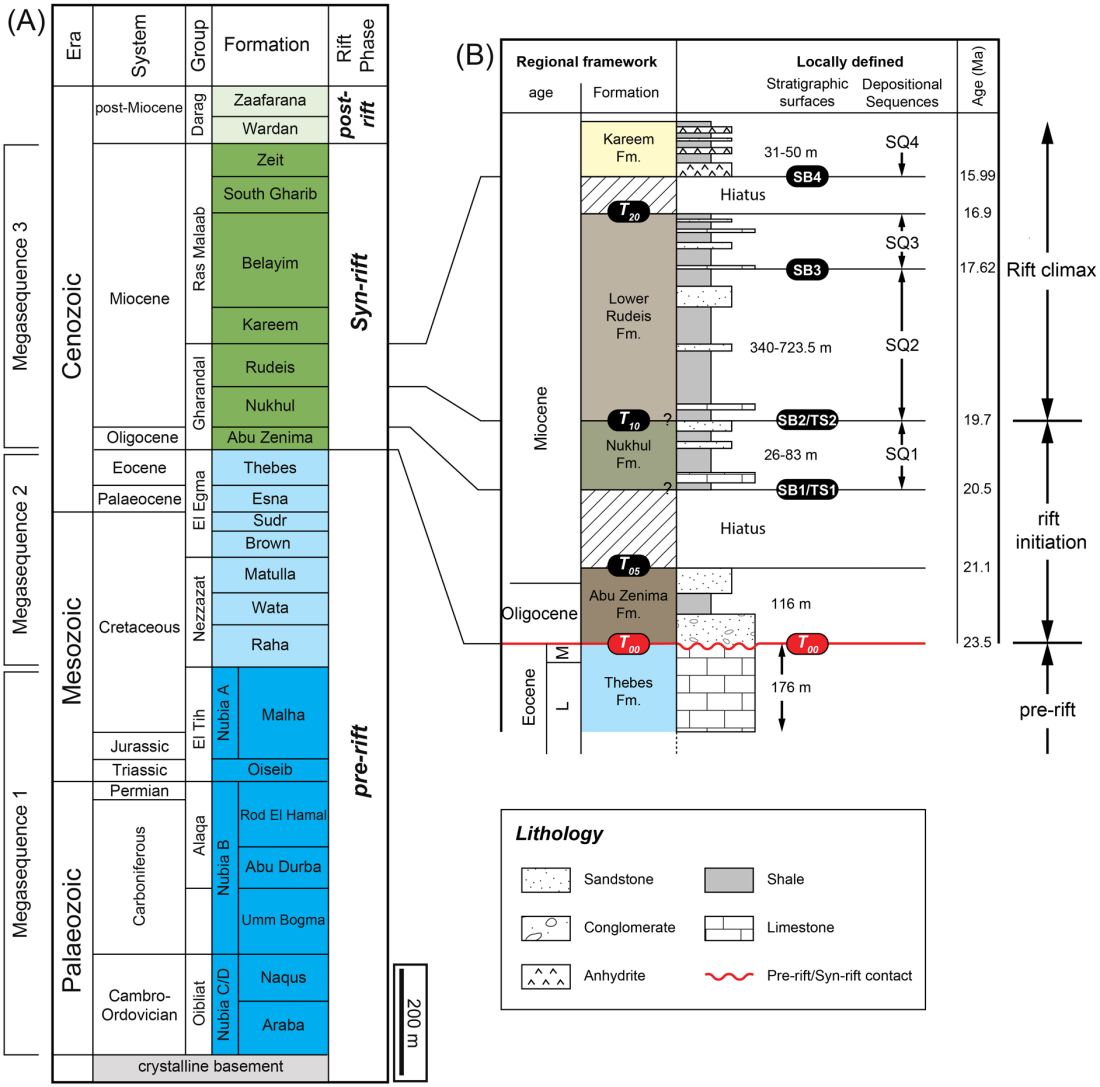
(**A**) Generalized stratigraphy of the Gulf of Suez Basin (after^53^). This study focuses on the early syn-rift Miocene sequence. (**B**) Local stratigraphic column showing the lithostratigraphy, key lithologies and sequence stratigraphic framework of the early Burdigalian to early Langhian succession in the Abu Rudeis–Sidri Field, east-central Gulf of Suez. Ages of key stratigraphic surfaces bounding early syn-rift units are also indicated (^54^ and present study).

The Gulf of Suez succession is traditionally divided into three tectonostratigraphic units—pre-rift, syn-rift, and post-rift strata, defined relative to the onset of Oligocene–Miocene rifting (e.g^42,50–52^.,; Fig. 2). The following description focuses on the Markha Plain, where formation thicknesses and facies are based on borehole ARM-7 and nearby wells. Only units intersected in the study interval are described, with emphasis on lithology, depositional environments, and tectonic significance relevant to Miocene hydrocarbon systems.

#### Pre-rift successions

Pre-rift successions are subdivided into two megasequences. Megasequence 1 comprises fluvial to shallow-marine sandstones of the Cambrian–Early Cretaceous Nubian Sandstone Group^55^, which unconformably overlie the Pan-African Precambrian basement. In the studied wells, however, only the Lower Cretaceous section was penetrated (~ 280 m), as drilling did not reach the basement. Megasequence 2 consists of a Late Cretaceous mixed carbonate–siliciclastic succession, the Raha, Wata, Matulla, Brown Limestone, and Sudr formations (~ 420 m), overlain by Paleocene-Eocene platform carbonates and shales of the Esna, Thebes, and related formations (~ 176 m; Fig. 2).

The entire pre-rift section, reaching a maximum thickness of ~ 1 km, is truncated by a regionally extensive latest Eocene–early Oligocene erosional surface that marks the onset of syn-rift deformation (^56^; Fig. 2). The studied half-graben system extends approximately 35–50 km in length, 10–15 km in width, and 3–4 km in depth, and is bounded by major listric normal faults.

#### Syn-rift successions

The syn-rift successions record a tectonically driven transgressive–regressive megasequence (Megasequence 3) associated with the evolution of the Gulf of Suez rift. Megasequence 3 begins with red-bed fanglomerates and sandstones of the Upper Oligocene–Lower Miocene Abu Zenima Formation (~ 116 m in well ARM-7), deposited during early fault-block exhumation under continental conditions. This is overlain by the Nukhul Formation (~ 31 m), composed of shallow-marine sandstones, marls, and calcareous conglomerates. Although commonly assigned an Aquitanian–Burdigalian age^57,58^, some microfossil data suggest a possible Chattian age, indicating ongoing uncertainty regarding its base^59–63^. Continued tectonic deepening during rift-climax subsidence led to deposition of the Rudeis Formation (~ 340 m), dominated by fine-grained marine mudstones and marls with minor intercalations of sandstone that were deposited during a rapid tectonic subsidence of the main rifting phase^4,64^. Up section, the succession shallows into the Kareem Formation (~ 172 m), characterized by fossiliferous shales and basal anhydrite, reflecting lagoonal to restricted-marine settings during a drop of relative sea-level. A progressive reduction in marine connectivity culminated in thick evaporite accumulation: the Belayim Formation (~ 400 m, anhydrite and halite), the South Gharib Formation (~ 501 m, massive halite and anhydrite), and the Zeit Formation (~ 452 m, halite with sandstone interbeds). The total syn-rift thickness in borehole ARM-7 is approximately 1.55 km. Stratigraphic nomenclature follows Egyptian General Petroleum Corporation^23^ and National Committee of Geological Sciences^24^ as shown in Table 1. The precise age of the Nukhul Formation is crucial not only for constraining the timing of rift initiation but also for applied purposes, including reservoir modeling, stratigraphic prediction, and analog selection for syn-rift reservoirs.

**Table 1:**
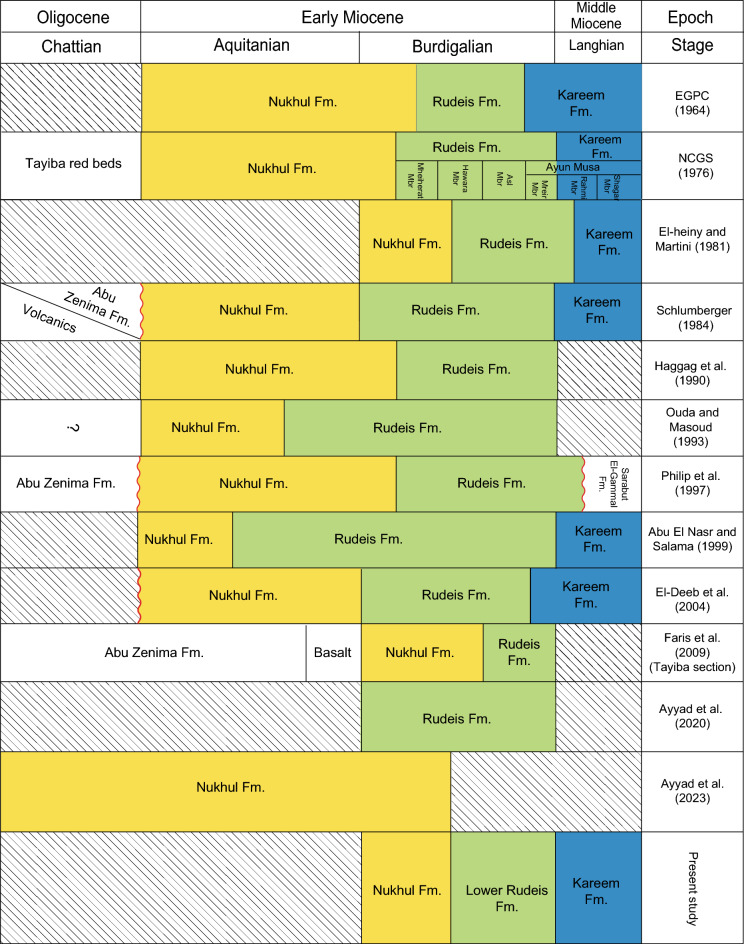
Comparison of the lithostratigraphic divisions of the Late Oligocene–Middle Miocene syn-rift succession in the Gulf of Suez Basin.

#### Post-rift successions

The post-rift successions of Pliocene–Quaternary age unconformably overlie the Miocene syn-rift sequence and exhibit significant lateral thickness variation. In borehole ARM-7, they reach a thickness of ~ 1.5 km and comprise gravels, sands, and minor evaporites of the Wardan and Zafarana formations (Fig. 2). These sediments were deposited in alluvial fans and fan-delta systems sourced from major wadis such as Feiran, Baba, and Sidri. They drape older tilted fault blocks and mark the final phase of basin infill.

## Materials and methods

The wireline-log data for four wells in the Abu Rudeis–Sidri Field, East Central Gulf of Suez, Egypt are available for the present study include ARM-7 (28° 51′ 43.05’' N – 33º 10′ 30.36’' E), ARS-6 (28° 51′ 20.23’' N – 33º 10′ 33.52’' E), SIDRI-20 (28° 50′ 53.2'' N – 33º 10′ 28.24’' E) and SIDRI-9 (28º 51′ 0.7'' N – 33º 11′ 15.23’' E). The biostratigraphic analysis was conducted on 129 ditch cutting samples collected from four onshore boreholes (ARM-7, ARS-6, SIDRI-9 and SIDRI-20) located in the central dip province of the Gulf of Suez. The two boreholes, ARM-7 and SIDRI-9, are nearly barren and contain very sparse faunal assemblages; consequently, only 86 out of 129 samples are considered valid. The dry rock samples (approximately 20 gm) were soaked in 10% H_2_O_2_ solution for 20 min for disaggregation. The samples were washed with distilled water through a 63 μm mesh sieve to remove clay fraction, and then dried in oven at 40 °C. This process enhances fossilization potential by removing juvenile foraminifera, which are difficult to identify^71^. The foraminifera were assembled using Olympus SZX7 binocular stereomicroscope. The foraminifera were investigated in detail with the JEOL JSM 6510 lv Scanning Electron Microscope (SEM) at Electron Microscope Unit, Mansoura University, Egypt.

**Fig. 3:**
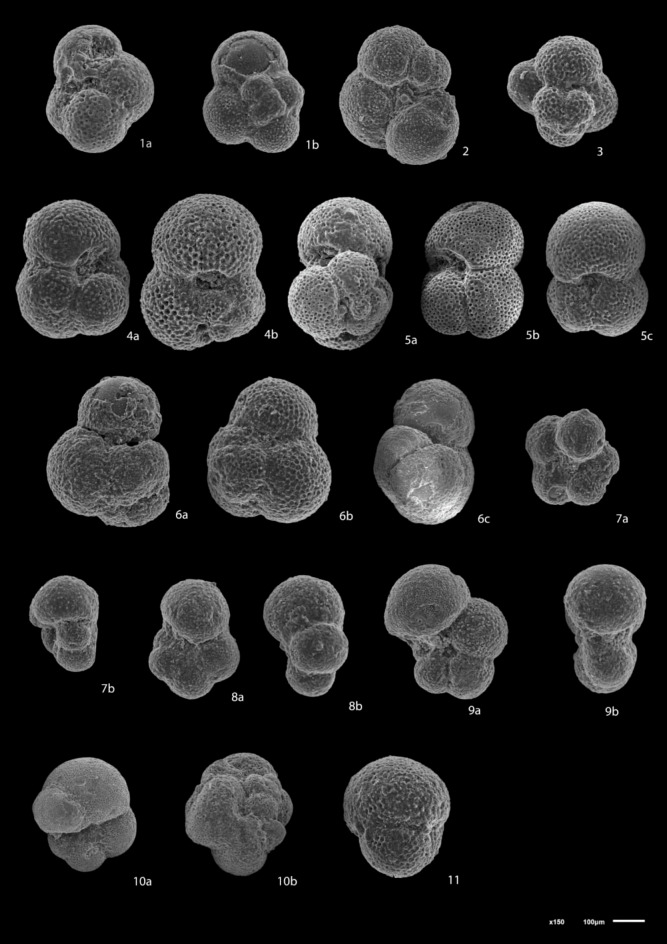
Scanning electron micrographs of selected planktonic foraminifera (scale bar = 100 μm). 1a, b: *Globigerina bulloides*, sample 25, Lower Rudeis Formation 2:* Globigerina falconensis*, sample 31, Nukhul Formation; 3: *Globigerinita uvula*, sample 6, Lower Rudeis Formation; 4a, b: *Globigerinoides quadrilobatus*, sample 4, Lower Rudeis Formation; 5a-c:*Trilobatus trilobus,* sample 4, Lower Rudeis Formation; 6a-c*: Globoturborotalita occlusa.*, sample 31, Nukhul Formation; 7a, b*: Paragloborotalia mayeri*, sample 13, Lower Rudeis Formation; 8a, b: *Paragloborotalia siakensis*, sample 33*,* Nukhul Formation; 9a, b*: Globogerinella obesa*, sample 12, Lower Rudeis Formation;, 10a, b: *Globorotaloides suteri *sample 4, Lower Rudeis Formation; 11: *Praeorbulina glomerosa*, sample 2, Kareem Formation.

**Fig. 4:**
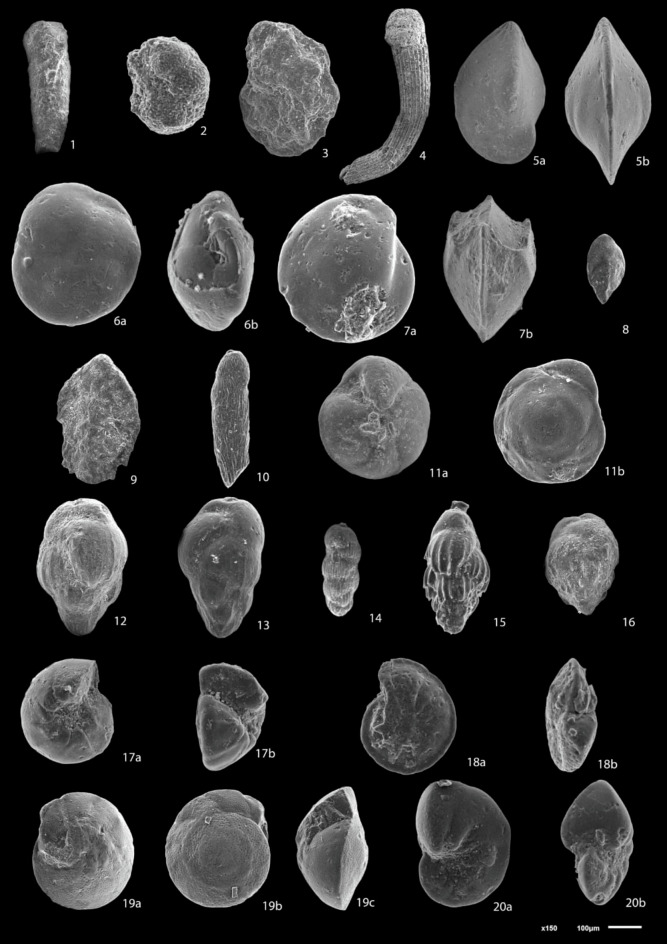
Scanning electron micrographs of selected benthic foraminifera (scale bar = 100 μm). 1: *Ammobaculites* sp., sample 23, Lower Rudeis Formation; 2: *Haplophragmides* sp*.*, sample 26, Lower Rudeis Formation: 3: *Cyclammina incisa*, sample 9, Lower Rudeis Formation; 4: *Nodosaria catenulata* var. *continuicosta*, sample 24, Lower Rudeis Formation; 5a, b: *Lenticulina budensis*, sample 7, Lower Rudeis Formation; 6a, b: *Lenticulina hughesi*, sample 16, Lower Rudeis Formation; 7a, b: *Lenticulina smileyi*, sample 40, Nukhul Formation; 8: *Bolivina fastigia*, sample 27, Lower Rudeis Formation; 9: *Bolivina superba*, sample 35, Nukhul Formation; 10: *Bolivina pseudospissa*, sample 10, Kareem Formation; 11a, b: *Globocassidulina monicana*, sample 2, Kareem Formation; 12: *Bulimina pupoides*, sample 40, Nukhul Formation; 13: *Buliminella curta*, sample 25, Lower Rudeis Formation; 14: *Buliminella subfusiformis*, sample 7, Lower Rudeis Formation; 15: *Uvigerina subperegrina, *sample 15, Lower Rudeis Formation; 16: *Uvigerina venusta*, sample 17, Lower Rudeis Formation; 17a, b: *Gyroidina* sp*.*, sample 33, Nukhul Formation; 18a, b: *Cibicides ellisi ellisi, *sample 40, Nukhul Formation; 19a, b: *Cibicidoides praecinctus*, sample 25, Lower Rudeis Formation; 20a, b: *Nonionella miocenica*, sample 29, Nukhul Formation.

The planktonic foraminiferal contents (Figs. 3, 4) obtained from the studied wells were identified using the^72^ monograph and analyzed quantitatively to make the biostratigraphic zonation following the biostratigraphic scheme of^73^ then determining the geological ages of the studied formations. The benthic foraminiferal assemblages were identified based on^74–77^and the systematic bases of^78^. Rotated Factor Analysis was performed using SPSS software to identify assemblages of benthic foraminifera. The abundances of foraminiferal species were counted for each sample. The paleoecological proxies for benthic foraminifera including Fisher’s α and Dominance have been estimated using the Paleontological.

Statistics Software Package (PAST) of^79^ to assess species diversity through time. Only species with a prevalence in excess of 5% have been considered.

Bathymetric zones adapted in the current study are based on^80^and^81^ as inner shelf (0–50 m), middle shelf (50–100 m), outer shelf (100–150 m) and upper slope (> 150 m). The paleobathymetric variations were quantitatively assessed using foraminiferal indices, containing the total foraminiferal number (TFN) and the planktonic/benthic (P/B) ratio (e.g^28,62,63,82^,). The spatial distribution of the identified benthic foraminiferal abundance in accordance with their depth-dependent depositional environments according to^83^ and^84^. Furthermore, the benthic foraminiferal species, percentage of Epifaunal taxa, the relative abundance of benthic foraminiferal genera and the ratio of agglutinated/calcareous benthic foraminifera are used in determination of paleoenvironmental settings.

Wireline log data includes gamma-ray, resistivity, neutron, density, and sonic logs across the four wells to provide lithological and reservoir property insights and support stratigraphic correlations. These logs are correlated between wells using Techlog® and Interactive Petrophysics (IP) software to underpin sequence stratigraphic interpretations. Sequence stratigraphic interpretations were conducted by analyzing seismic data and wireline logs, based on the sequence stratigraphic framework presented here, which is founded on the recognition of key stratal surfaces following sequence stratigraphic principles^85–94^. Seismic interpretation of 18 seismic sections, performed using Petrel™ software, included identifying key stratal surfaces and fault patterns to establish timelines. These surfaces were correlated with wireline log data to recognize systems tracts and depositional sequences across shallow-marine and shelf settings. The conventional ditch samples were fitted with their log-responses. The MFS is indicated by peaks in the abundance of planktonic foraminifera and the infaunal benthic taxa such as *Uvigerina* and *Bulimina*^95–97^.

## Results

### Lithostratigraphy

Three lithostratigraphic units are recognized in ascending order in the studied boreholes: the Lower Miocene Nukhul and Lower Rudeis formations, and the Middle Miocene Kareem Formation (Table 1). These formations are assigned to the Gharandal Group^23,98,99^.

#### The nukhul formation

**Fig. 5:**
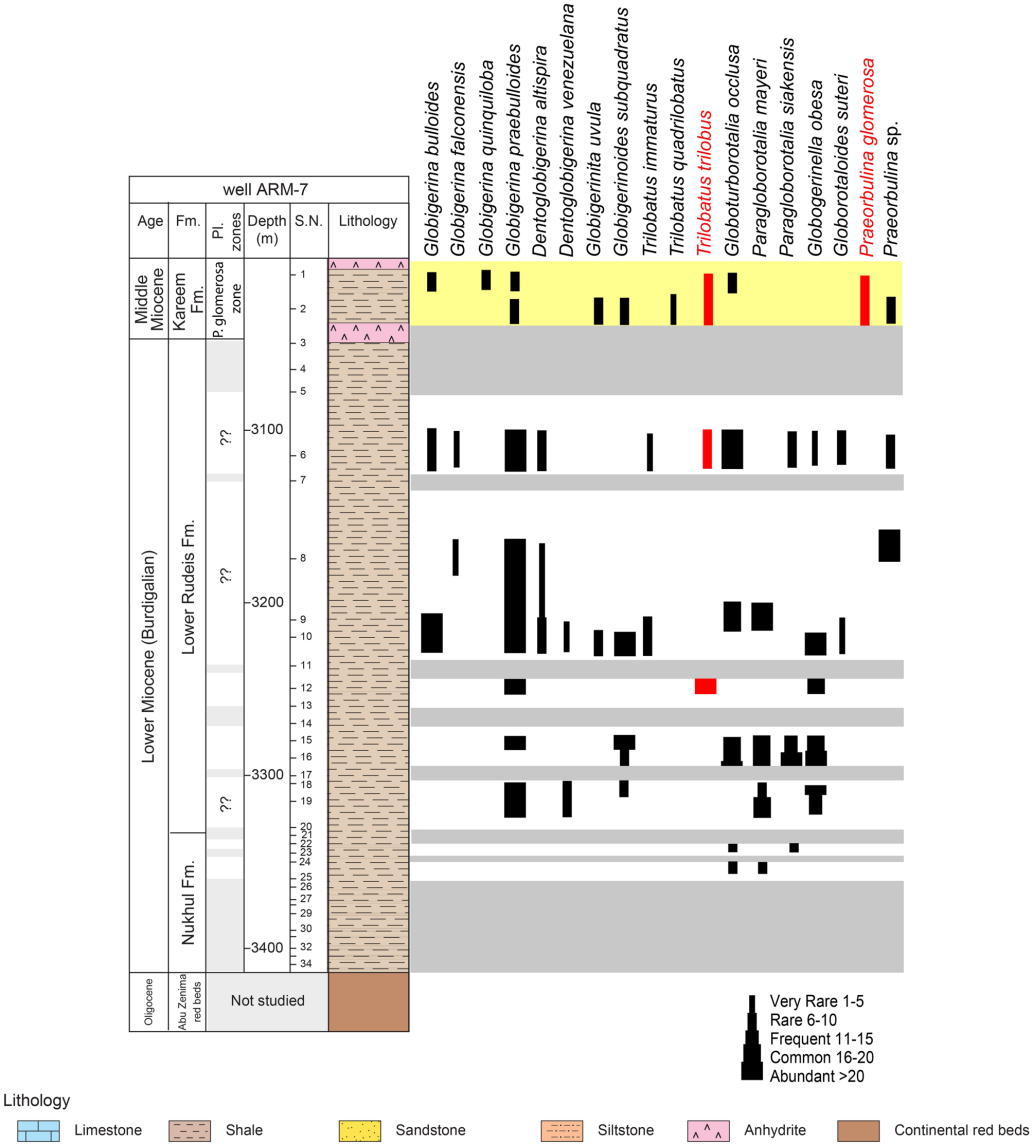
Distribution chart of identified planktonic foraminifera relative abundance in well ARM-7 against lithostratigraphy.

**Fig. 6:**
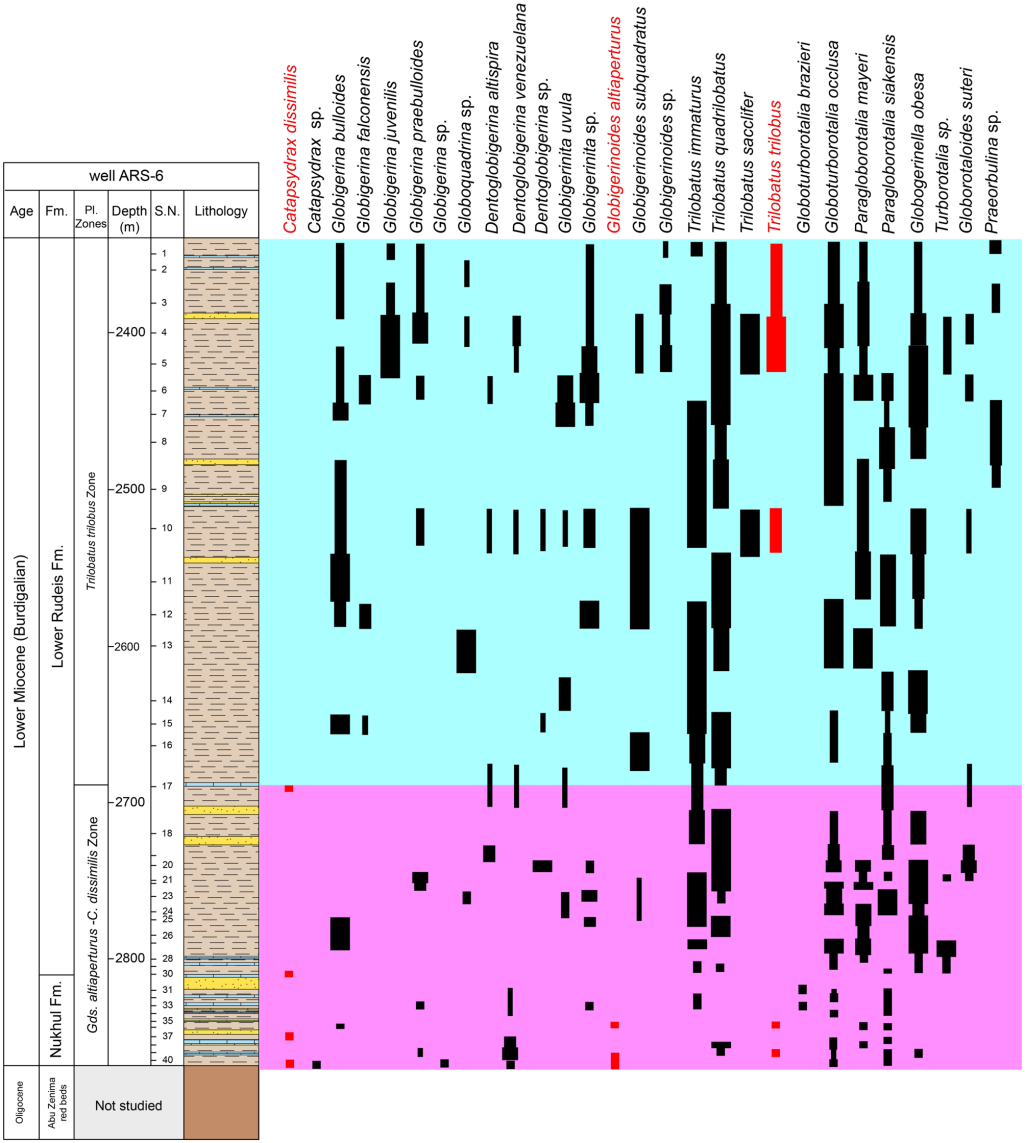
Distribution chart of identified planktonic foraminifera relative abundance in well ARS-6 against lithostratigraphy and wireline-log trends.

**Fig. 7:**
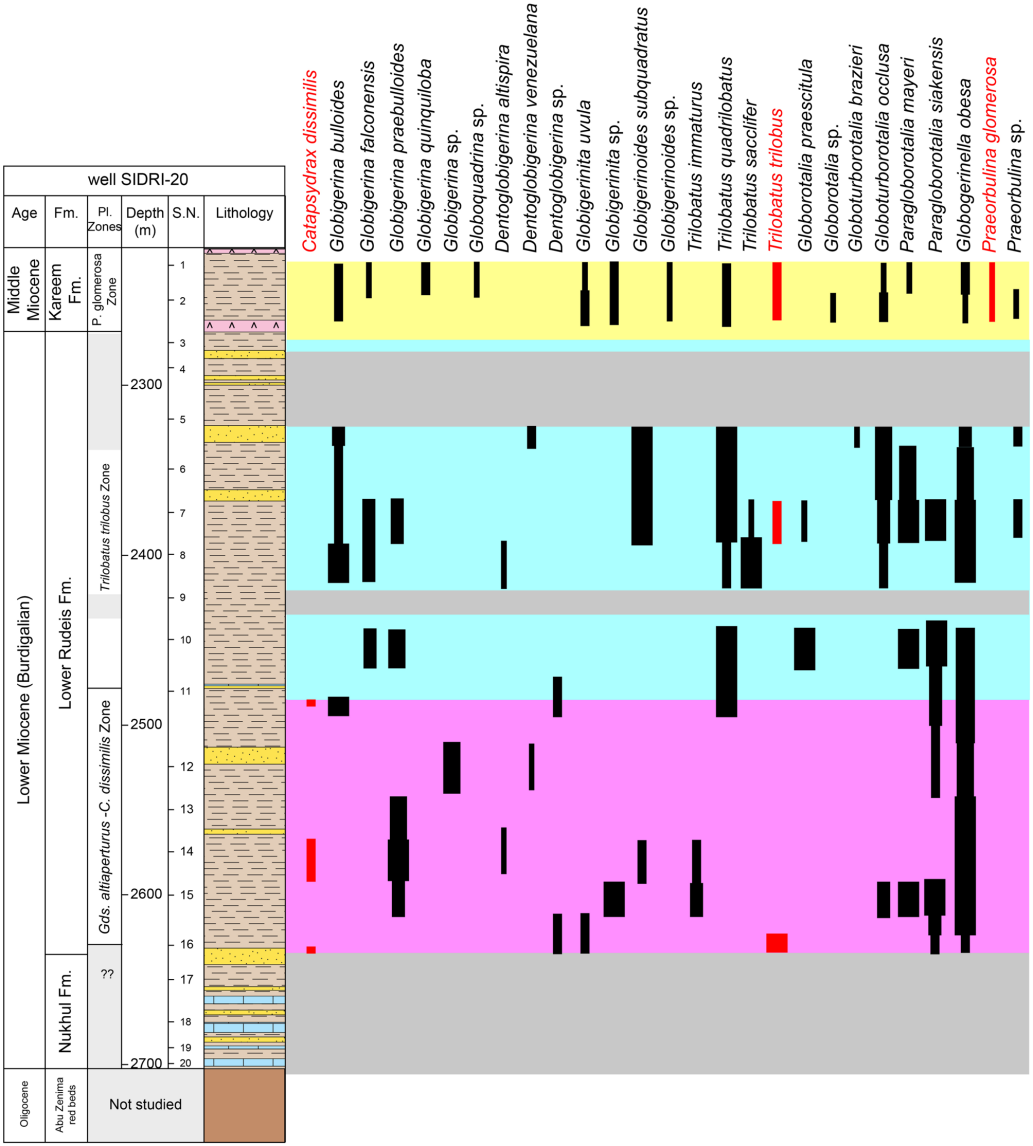
Distribution chart of identified planktonic foraminifera relative abundance in well SIDRI-20 against lithostratigraphy and wireline-log trends.

**Fig. 8:**
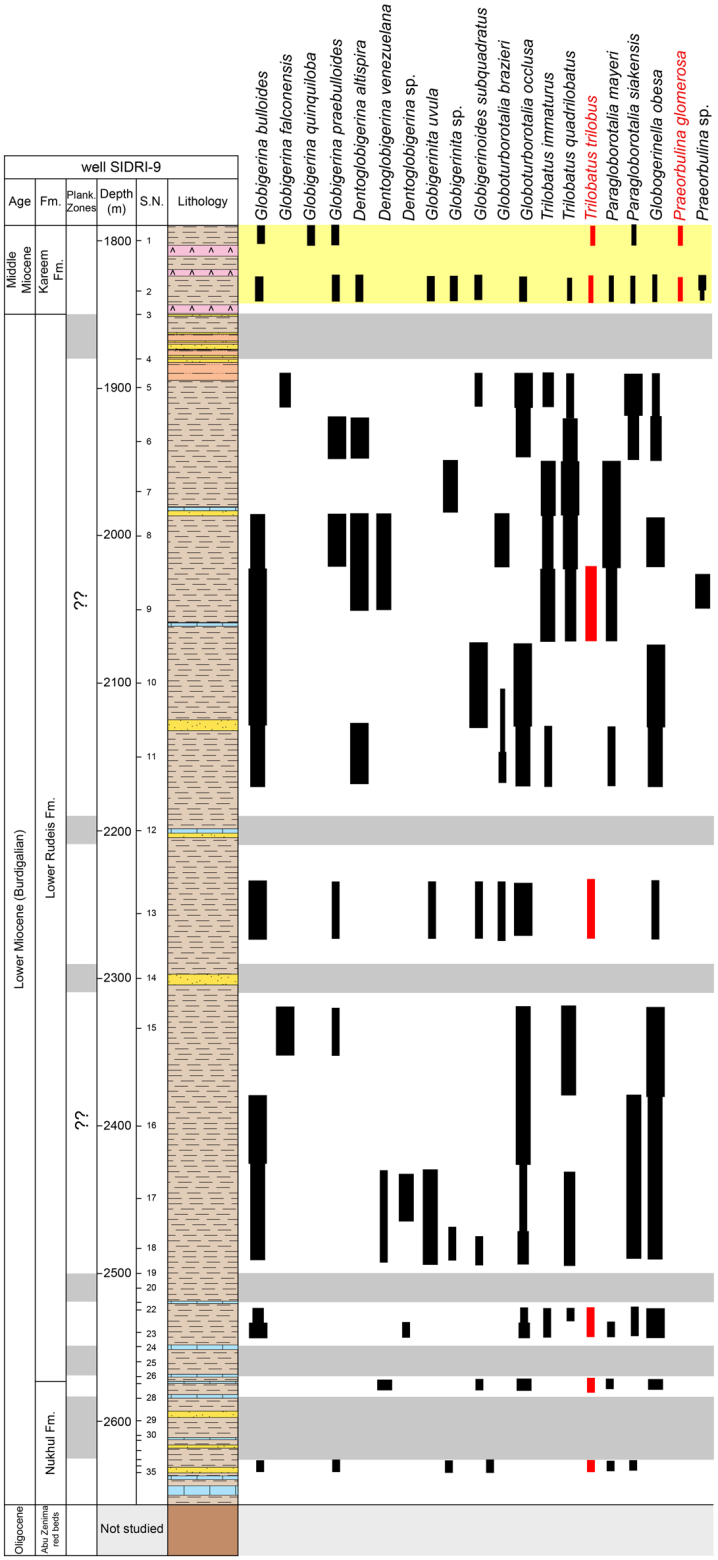
Distribution chart of identified planktonic foraminifera relative abundance in well SIDRI-9 against lithostratigraphy.

The Nukhul Formation is penetrated in all four boreholes. Examination of composite logs shows that the Nukhul Formation rests unconformably on the Late Oligocene Abu Zenima Formation across a stratigraphic hiatus exceeding 2.5 Ma (Figs. 5, 6, 7, 8) and is, in turn, unconformably overlain by the Early Miocene Rudeis Formation. Thickness increases southeastward from 23 m in ARM-7 to 83 m in SIDRI-9. Lithologically, the unit comprises gray to dark-gray, highly calcareous shales interbedded with fine- to medium-grained calcareous sandstones and thin limestone layers. Shales are soft to moderately hard, sub-blocky to flaky, and locally glauconitic or argillaceous; sandstones are sub-rounded to rounded, moderately sorted, commonly oil-stained; limestone lenses are cryptocrystalline to fine-crystalline, creamy white to light brown, and locally sandy or argillaceous.

**Fig. 9:**
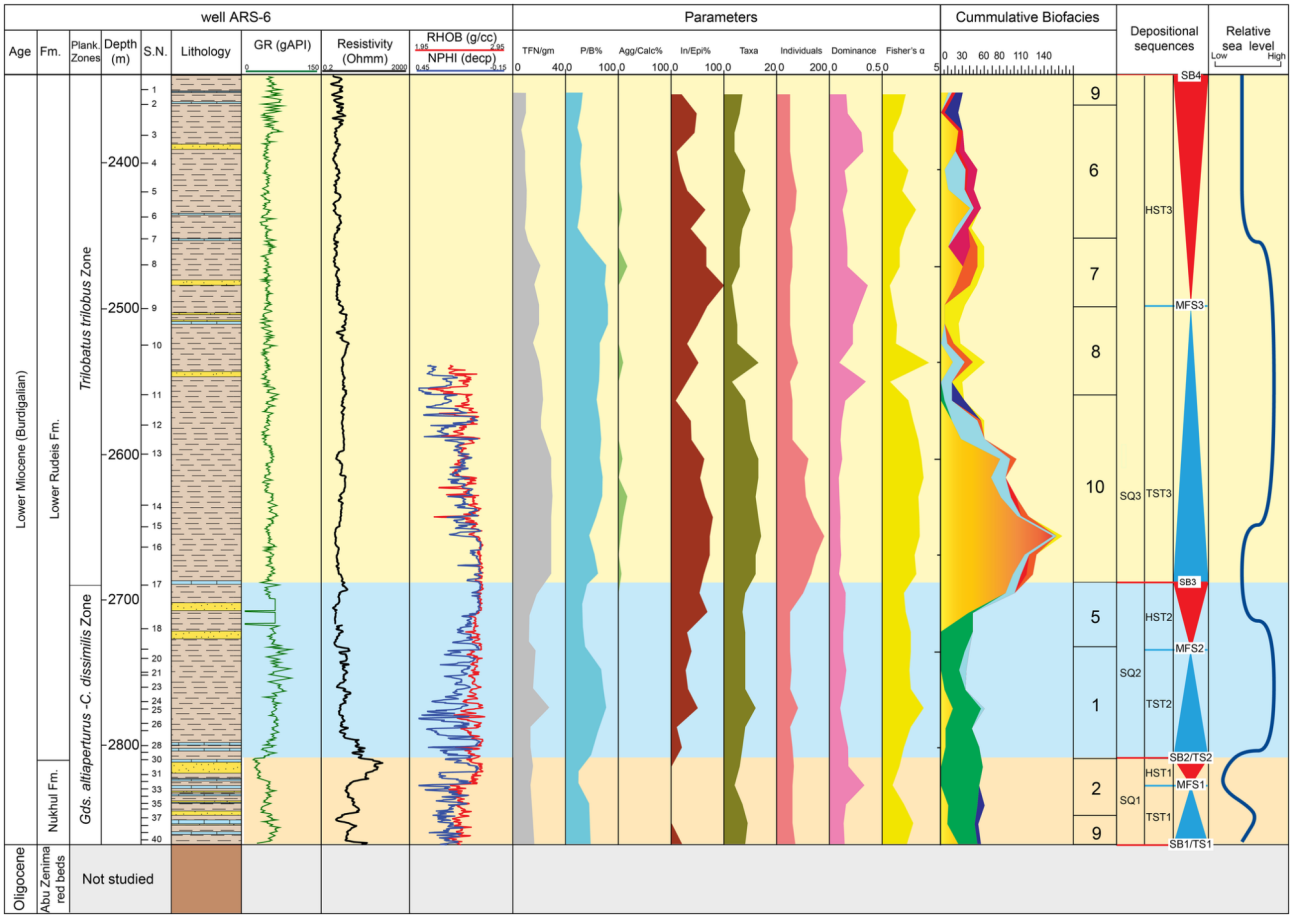
Distribution of the recognized benthic foraminiferal biofacies in well ARS-6 against wireline-log trends, applied foraminiferal indices, and sequence stratigraphic framework. TFN = Total Foraminiferal Number; P/B% = Planktonic/Benthic foraminiferal ratio; Agg/Calc% = Agglutinated/Calcareous ratio; In/Epi% = Infaunal/Epifaunal ratio.

**Fig. 10:**
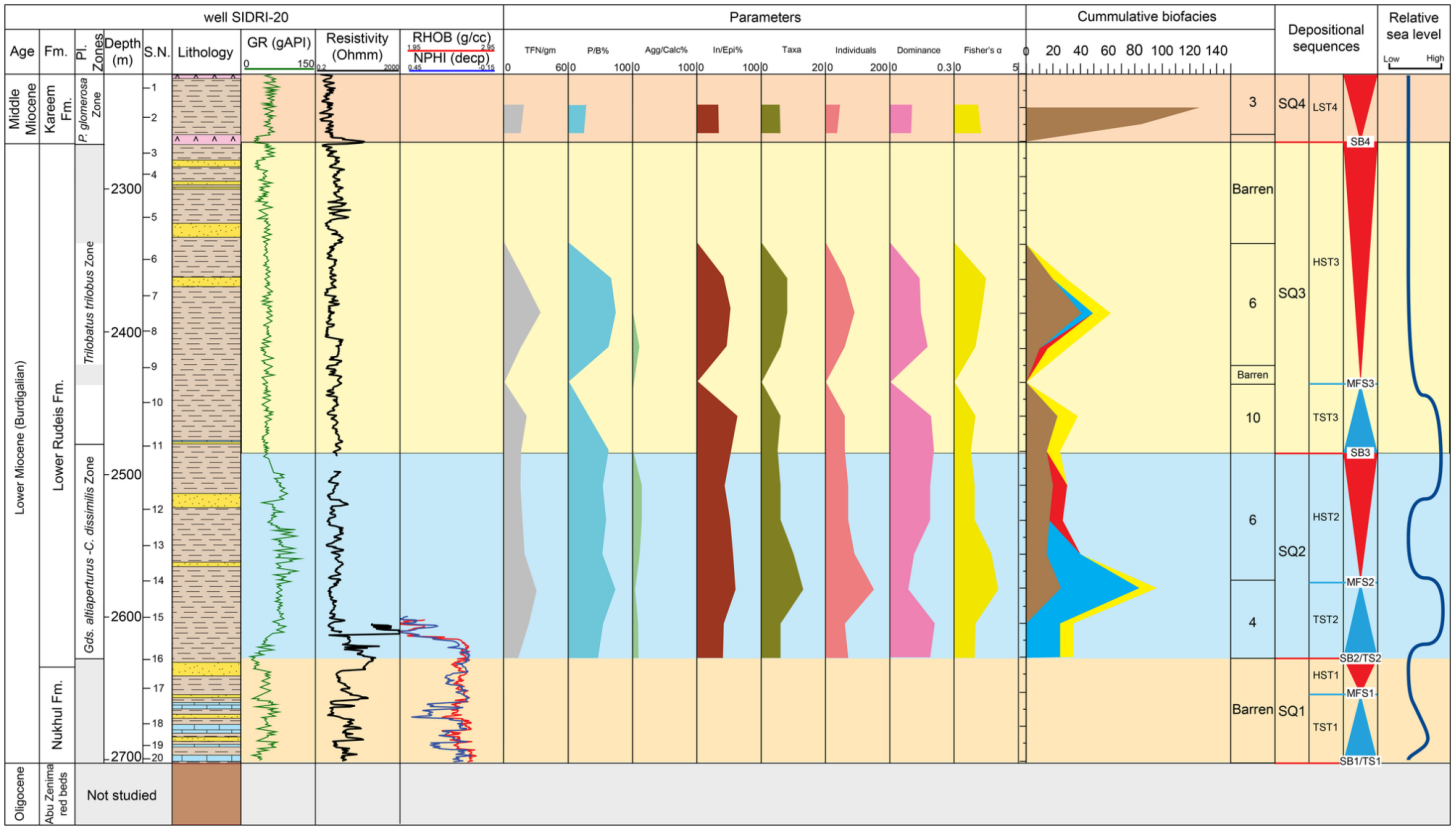
Distribution of the recognized benthic foraminiferal biofacies in well SIDRI-20 against wireline-log trends, applied foraminiferal indices, and sequence stratigraphic framework. TFN = Total Foraminiferal Number; P/B% = Planktonic/Benthic ratio; Agg/Calc% = Agglutinated/Calcareous ratio; In/Epi% = Infaunal/Epifaunal ratio.

**Fig. 11:**
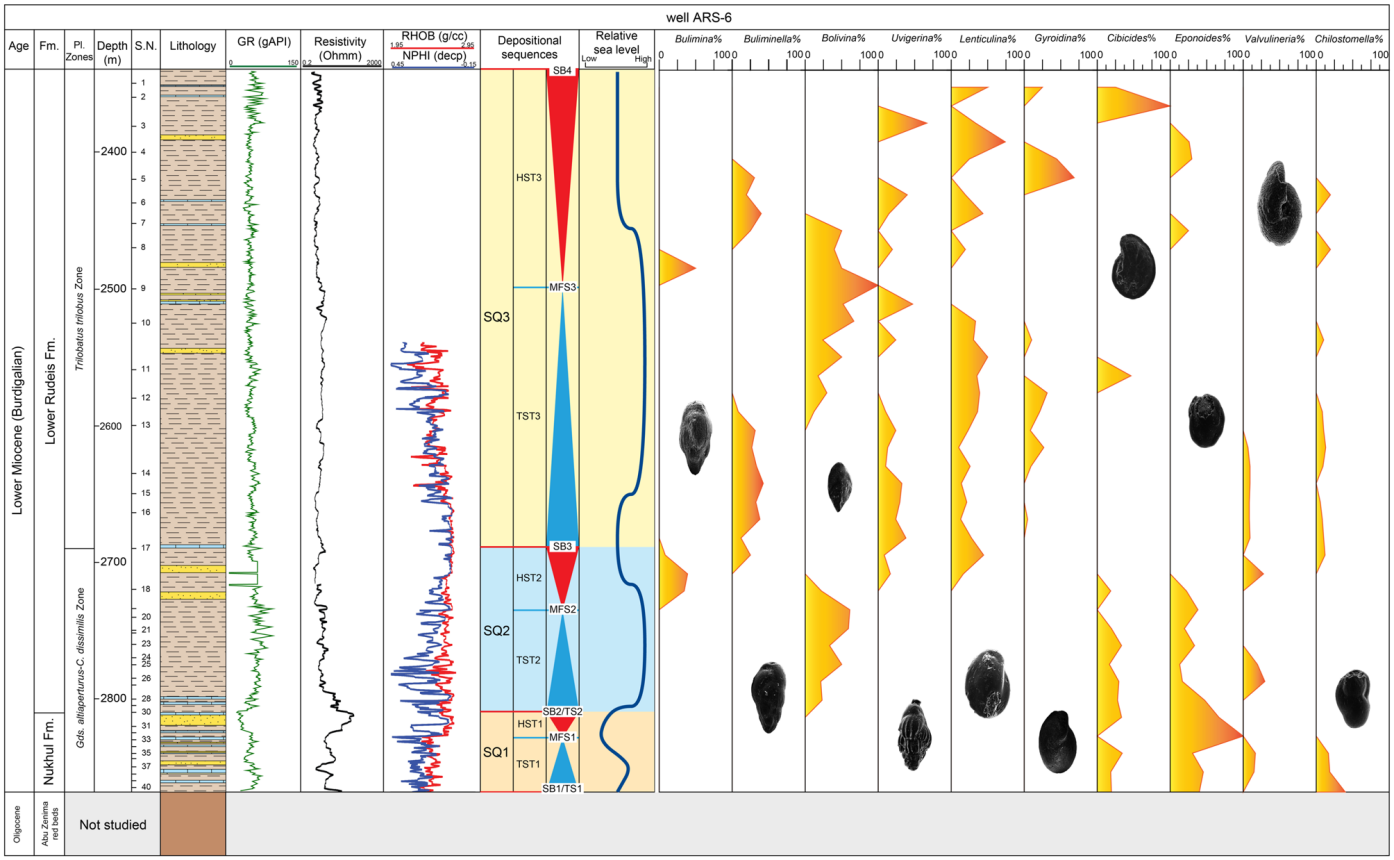
Relative abundance of the dominant benthic foraminiferal genera, wireline-log trends, and sequence stratigraphic framework in well ARS-6.

**Fig. 12:**
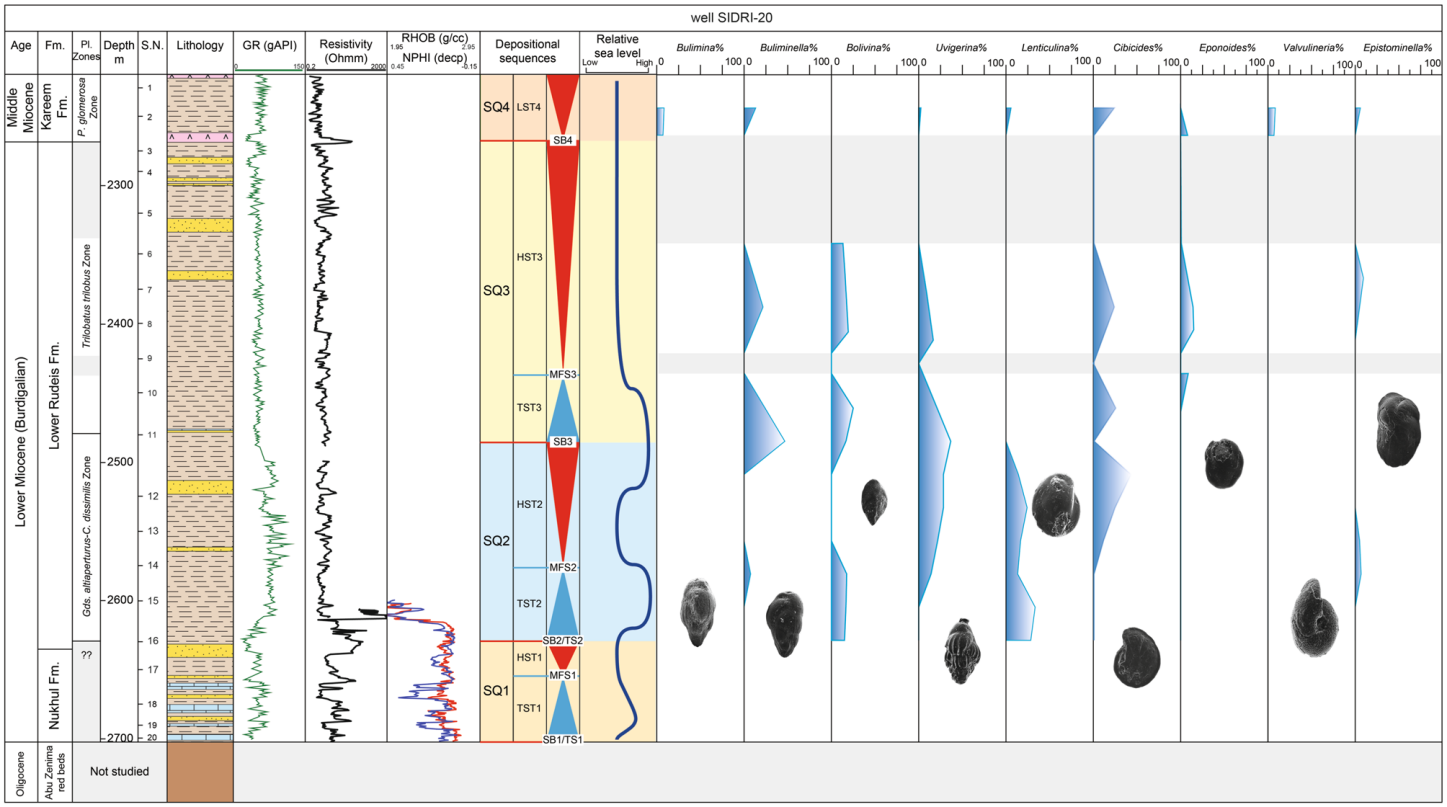
Relative abundance of the dominant benthic foraminiferal genera, wireline-log trends, and sequence stratigraphic framework in well SIDRI-20.

In the Abu Rudeis–Sidri Field, Nukhul sandstones form the principal reservoir, exhibiting porosities of 10–16% and water saturation below 34%^100^. A shallow-marine inner-shelf setting is inferred from vertical facies stacking and the dominance of shallow-water benthic assemblages (*Bolivina*, *Nonion*, *Cibicides*) shown in Fig. 9, together with low to moderate gamma-ray values and lithofacies transitions integrated in Figs. 10, 11, 12.

#### The rudeis formation

The Rudeis Formation, dated to the Burdigalian–Langhian^101^, rests unconformably on the Nukhul Formation and is itself unconformably overlain by the Kareem Formation, where the intra-Burdigalian (Mid-Rudeis) and base-Kareem unconformities merge. The Mid-Rudeis event marks a fundamental change in basin geometry, renewed extensional reactivation with fault-block rotation, and accelerated uplift of the rift margins; consequently, the Lower Rudeis was deposited during the rift-climax phase under high subsidence rates^42^.

The unit is present in all four boreholes, thickening from 340 m in ARM-7 to 723.5 m in SIDRI-9. Regionally the Rudeis is subdivided into Lower and Upper members, but only the Lower Rudeis (equivalent to the Mheiherrat Formation elsewhere in the Gulf) is preserved here. Lithology is dominated by gray to greenish-gray, highly argillaceous, calcareous shales, interbedded with fine- to medium-grained, off-white to yellow sandstones, siltstones, and thin limestones; shales are locally glauconitic and vary from soft to hard, while sandstones are moderately sorted and calcareous.

These facies record progressive deepening from middle shelf to upper-slope settings. Evidence includes high gamma-ray values and the appearance of deep-water benthic taxa such as *Buliminella*, *Uvigerina*, *Lenticulina*, and *Gyroidina* (Fig. 9), together with the integrated gamma-ray lithofacies profiles in Figs. 10, 11, 12. The basal transgressive surface (T10, ~ 19.7 Ma^54^;) underlies this deepening trend, while the top is truncated by the regional T20 unconformity (~ 16.9 Ma), generated during uplift and block rotation at rift climax^42^.

#### The kareem formation

The available data for the Kareem Formation in the Abu Rudeis–Sidri Field is particularly limited and pertains only to its lower interval. The Kareem Formation rests unconformably atop the Lower Rudeis Formation, with the base marked by the merged Mid-Rudeis and base-Kareem unconformities. The Kareem Formation, of Langhian–Early Serravallian age^23,66^, is recognized in three boreholes (ARM-7, SIDRI-9, and SIDRI-20), but is absent in ARS-6. Its thickness varies from 31 to 50 m and increases toward the south and southeast. The unit consists of fossiliferous shales interbedded with anhydrite and thin sandstone beds. The anhydrite is white to milky, fine- to medium-grained, soft to moderately hard, and cryptocrystalline; shale is greenish-gray to light gray, hard, sub-blocky to flaky, glauconitic, fossiliferous, and non-calcareous to calcareous. The sandstones are white, loose, fine- to medium-grained, subrounded to subangular, and calcareous cemented. A restricted inner-shelf lagoonal environment is inferred from the laminated shale–anhydrite succession and the dominance of shallow benthic taxa such as *Cibicides*, *Epistominella*, and *Quinqueloculina* (Fig. 10). This interpretation is supported by lithofacies patterns and gamma-ray profiles in Figs. 10, 11, 12, indicating a shift from open marine to restricted conditions during post-climax tectonic uplift and relative sea-level fall^42,50^.

### Biostratigraphy

The Miocene biostratigraphy is established using planktonic foraminiferal biozonation and benthic foraminiferal biofacies analysis. The foraminiferal assemblages obtained from the studied succession comprise 11,970 specimens, including 4,050 benthic and 7,920 planktonic foraminifera. The recorded foraminiferal assemblages in the studied boreholes include thirty-two planktonic foraminiferal species belonging to fourteen genera, forty-eight benthic foraminiferal species belonging to twenty-eight genera that enables the identification of three planktonic foraminiferal biozones. The absence of characteristic planktonic species *Globigerinoides primordius* may be attributed to a significant hiatus of Aquitanian age, resulting from tectonic-related unconformity. The most significant planktonic and benthic foraminiferal species are illustrated in Figs. 3, 4.

#### Planktonic foraminiferal biozones

Biostratigraphic analysis is based on recognition of planktonic foraminiferal assemblages used to establish biozones and their likely tie to inter-regional and global planktonic biozonation schemes (Table 2). The vertical distributions of planktonic foraminiferal species enable the recognition of two planktonic biozones: *Globigerinoides altiaperturus- Catapsydrax dissimilis* Subzone and *Trilobatus trilobus* Zone in well ARS-6, three biozones: *Globigerinoides altiaperturus-Catapsydrax dissimilis* Zone, *Trilobatus trilobus* Zone and *Praeorbulina glomerosa* Zone in well SIDRI-20 and one biozone *Praeorbulina glomerosa* Zone in wells ARM-7 and SIDRI-9 (Figs. 5–8). These zones do not correlate with the global biozonation schemes of^102^ and^103^due to the absence of their endemic marker species and biozones and tectonic-related unconformities that disrupted the stratigraphic continuity in the Gulf of Suez. For example, the absence of *Globigerinoides sicanus*, a well-established biostratigraphic marker in both surface and subsurface sections of the Gulf of Suez, is noteworthy. In particular, the Nukhul and Rudeis formations exhibit evidence of episodic rifting and uplift, which led to intermittent basin restriction and periods of reduced water depth, especially during the early stages of rifting. This led to the absence or rarity of global deep-water marker taxa, as noted in prior studies^29,76,104,105^, resulting in significant erosional hiatuses. Consequently, several authors have proposed local biostratigraphic zonal schemes based on planktonic foraminifera (e.g^26,28,73^.,) to accommodate these gaps, necessitating a regionally adjusted framework tailored to the tectonic disruptions observed in the studied wells. The age of the studied rock units is constrained by calcareous nannoplankton analysis from selected samples (ARM-7: 24, 22, 16, 12, 8, 2, 1; ARS-6: 38, 35, 32, 29; SIDRI-20: 17, 16, 12, 7, 6, 2, 1; SIDRI-9: 27, 23, 9, 8). The Nukhul and Rudeis formations are assigned a Burdigalian age corresponding to the NN4 biozone, characterized by an assemblage comprising *Helicosphaera ampliaperta*, *Sphenolithus heteromorphus*, *Helicosphaera carteri*, *H. ampliaperta*, *H. kamptneri*, *Discoaster variabilis*, *D. druggii*, *D. deflandrei*, *Cyclicargolithus floridanus*, and *Coccolithus pelagicus*. The overlying Kareem Formation is assigned to the NN5 biozone, marked by an assemblage including *Sphenolithus heteromorphus*, *Helicosphaera carteri*, *H. kamptneri*, *Coccolithus miopelagicus*, *Cyclicargolithus floridanus*, and *Discoaster druggii*, indicative of a Langhian age.

**Table 2:**
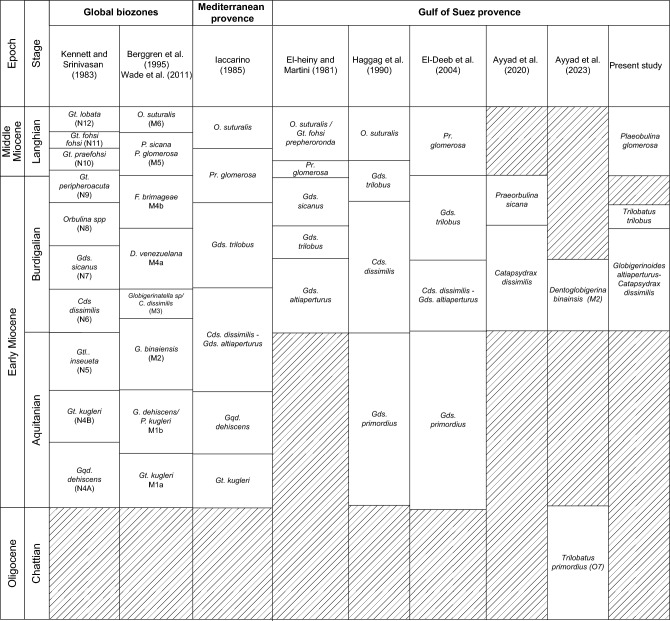
Comparison of the planktonic foraminiferal zones of the Late Oligocene-Middle Miocene syn-rift succession in the Gulf of Suez Basin, Mediterranean province and global equivalents.

The correlation between the identified foraminiferal biozones and their counterparts in Egypt is shown in Table 2. We relied on the zonal scheme of^73^ in defining the foraminiferal zones as the studied interval boundaries are highly fitted. The biostratigraphic analysis of the Miocene strata from the Gulf of Suez has assigned the Nukhul Formation to Burdigalian age (^26,70,106^ “Wadi Tayiba section”). The Nukhul Formation is reported here as being of Burdigalian age based on the major unconformity at the base of Nukhul Formation and the absence of key planktonic species from the Aquitanian age. The base of the Burdigalian age in this region is defined by the lowest occurrence (LO) of *Globigerinoides altiaperturus*, as established by^102,103^, and^107^.

The Early–Middle Miocene boundary corresponds to the base of the Langhian stage^102,108^. In this context, the Burdigalian-Langhian boundary is placed between the Lower Rudeis and Kareem formations^27,68,69,107,109^. The identified planktonic zones in the studied boreholes are briefly outlined below, from the oldest to the youngest:

##### Globigerinoides altiaperturus-Catapsydrax dissimilis Subzone

This subzone is reported by^73^ as being of Burdigalian age. It is characterized by the lowest occurrence (LO) of *Globigerinoides altiaperturus* and the highest occurrence (HO) of *Catapsydrax dissimilis*. This subzone is present within the Nukhul Formation and the lower part of the Lower Rudeis Formation in well ARS-6, at depth ranging from 2865 to 2690 m. It is also detected in the well SIDRI-20 to depth of 2480 m and its base is not defined due to barren interval and is not found at the two other wells due to many barren intervals (Figs. 6, 7). It corresponds to the *Globigerinoides altiaperturus-Catapsydrax dissimilis* Zone identified by^65^, the *Globigerinita dissimilis- Globigerinoides altiapertura* Zone (N5-N6) of^106^ and *Globiginantella insueta-Catapsydrax dissimilis* Zone of^102^. Additionally, it is equivalent to the *Catapsydrax dissimilis* Zone established by^110^, and the *Globigerinatella* sp.*- Catapsydrax dissimilis* Zone of^103^. Its characteristic foraminiferal assemblage includes* Globigerinoides altiaperturus*, *Catapsydrax dissimilis*, *Trilobatus immaturus*, *Trilobatus quadrilobatus*, *Trilobatus trilobus*, *Globoturborotalia occlusa*,* Globigerina bulloides*,* Globigerina praebulloides*, *Globigerinita uvula*, *Paragloborotalia mayeri*, *Globogerinella obesa* and *Paragloborotalia siakensis*.

###### Remarks:

This zone was initially introduced by^111^ and later designated as a subzone termed the *Globoquadrina dehiscens l Catapsydrax dissimilis* Zone by^112^ and^73^. This zone is divided into two subzonesin ascending order: the *Globoquadrina dehiscens dehiscens-Globigerinoides altiaperturus* Subzone of Aquitanian age and the *Globigerinoides altiaperturus l Catapsydraxdissimilis* Subzone of Burdigalian age. In this study, the first subzone was not assigned because *Globoquadrina dehiscens* was absent. the first subzone was not designated because of the absence of *Globoquadrina dehiscens*. The marker species of *Catapsydrax dissimilis* and *Globgerinoides altiaperturus* are infrequent and exhibit sporadic occurrences.

##### Trilobatus trilobus Interval Zone

This zone is classified as belonging to the Burdigalian age (Early Miocene) according to^73^ and was initially introduced by^111^. It is defined by the HO *Catapsydrax dissimilis* to LO of *Praeorbulina glomerosa*. The *Catapsydrax dissimilis* begins in the Eocene and it is very important for its highest occurrence in the Early Miocene^73^. This zone encompasses the biostratigraphic interval of the upper part of the Lower Rudeis in well ARS-6, extending from a depth of 2690 m with an undefined upper limit, and from 2480 to 2269 m in well SIDRI-20 (Figs. 6, 7). It is not found at the two other wells due to many barren intervals (Figs. 5, 8). It is equivalent to the *Globigerinoides trilobus* Zone of^26,65^ and^110^. Also, it corresponds to the *Globigerinoides triloba* Zone (N7) described by^106^, and the *Globigerinoides bisphericus* Zone of^103^. It is characterized by the presence of* Globigerines* assemblage, *Globigerinita uvula*, *Globoturborotalia occlusa*, *Globigerinoides subquadratus*, *Trilobatus sacclifer Trilobatus quadrilobatus*, *Trilobatus trilobus*, *Paragloborotalia mayeri*, *Globogerinella obesa* and *Paragloborotalia siakensis*.

##### Praeorbulina glomerosa lineage zone

This zone has been reported by^73^ as being of Langhian age, and it was originally introduced by^111^. It is defined by the LO of *Praeorbulina glomerosa* to LO of *Orbulina suturalis.* This zone is recognized only in the lower part of the Kareem Formation across the three studied wells, with depths ranging from 3330 to 3310 m in well ARM-7, 2250 to 2230 m in well SIDRI-20, and 1834 to 1800 m in well SIDRI-9. It is not defined in well ARS-6 as the Kareem Formation is missing (Figs. 5, 6, 7, 8). It corresponds to the *Praeorbulina glomerosa* Zone of^112^, *Praeorbulina glomerosa* Zone (N8) of^26,65,106^ and^68^. Additionally, it is equivalent to the *Praeorbulina sicanus* Zone of^29,105,113^ and^67^. Its characteristic foraminiferal assemblage includes* Globigerina quinquiloba*, *Globoturborotalia occlusa*, *Trilobatus quadrilobatus*, *Trilobatus trilobus*, *Paragloborotalia mayeri*, *Globogerinella obesa*, *Paragloborotalia siakensis* and *Praeorbulina glomerosa.*

#### Benthic foraminiferal biofacies

Benthic foraminifera represent a valuable tool for paleobathymetric reconstruction and have significant utility in paleoenvironmental interpretation. In this study, ten benthic foraminiferal biofacies are identified based on the consistent occurrence and vertical distribution of foraminiferal species and associated fauna (Figs. 9, 10; Table 3). Cumulative biofacies: the numbers of benthic foraminiferal spp. in each cluster are combined in one column (Figs. 9, 10).

**Table 3:** Statistically recognized benthic foraminifera biofacies and their interpreted paleoenvironmental settings.

Biofacies	Species	Oxygenation	Life Habitat	Diversity		P/B %	Calc./Aggl	Environmental Condition	Paleoenvironment
				Fisher’s α	Dominance				
w1	*Bathysiphon taurinensis*	Dysoxic	Epifaunal/shallow infaunal	2.04 to 3.9	0.11 to 0.15	50 to 80	Aggl.	Low oxic	Outer shelf to upper slope (<200 m)
	*Dentalina baggi*	Dysoxic	Infaunal				Calc.		
	*Nodosaria catenulata var.continuicosta*	Suboxic	Epifaunal/shallow infaunal				Calc.		
	*Lenticulina smileyi*	Oxic	Epifaunal				Calc.		
	*Bulimina pupoides*	Dysoxic	Infaunal				Calc.		
	*Buliminella elegantissima*	Dysoxic	Infaunal				Calc.		
	*Buliminella subfusiformis*	Dysoxic	Infaunal				Calc.		
	*Uvigerina senticosa*	Dysoxic	Infaunal				Calc.		
	*Uvigerina venusta*	Dysoxic	Infaunal				Calc.		
	*Valvulineria minuta*	Dysoxic	Epifaunal				Calc.		
	*Chilostomella ovoidea*	Oxic/dysoxic	Epifaunal/shallow infaunal				Calc.		
2	*Bolivina dilatate*	Dysoxic	Infaunal	0.1 to 2	0.1 to 0.12	10 to 25	Calc.	Dysoxic to oxic	Inner shelf (0–50 m)
	*Bolivina superba*	Dysoxic	Infaunal				Calc.		
	*Bulimina *sp1	Dysoxic	Infaunal				Calc.		
	*Eponoides *sp	Oxic	Epifaunal				Calc.		
	*Discorbis obtuse*	Oxic	Epifaunal				Calc.		
	*Cibicides dutemplei*	Oxic	Epifaunal				Calc.		
	*Nonion scapha*	Suboxic	Epifaunal/shallow infaunal				Calc.		
	*Pseudononion basispinata*	Suboxic	Epifaunal/shallow infaunal				Calc.		
3	*Quiquiloculina seminula*	Oxic	Epifaunal	0.2 to 1.5	0.09–0.1.09.1	10 to 15	Calc.	Oxic to low dysoxic	Shelf lagoon (0–5 m)
	*Spiroloculina tenuis*	Oxic	Epifaunal				Calc.		
	*Cibicides praecinctus*	Oxic	Epifaunal				Calc.		
	*Epistominella smithi*	Oxic	Epifaunal				Calc.		
4	*Ammobaculites *sp	Dysoxic	Epifaunal/shallow infaunal	1.5 to 3.4	0.11 to 0.2	46 to 72	Aggl.	Low oxic	Middle to outer shelf (50-150m)
	*Nodosaria ovicula*	Suboxic	Epifaunal/shallow infaunal				Calc.		
	*Loxostomoides digitata*	Dysoxic	Epifaunal/shallow infaunal				Aggl.		
	*Bolivina conica*	Dysoxic	Epifaunal/shallow infaunal				Calc.		
	*Nonionella miocenica*	Dysoxic	Epifaunal/shallow infaunal				Calc.		
5	*Lenticulina budensis*	Oxic	Epifaunal	2.9 to 3.4	0.11 to 0.14	60 to 80	Calc.	Suboxic to oxic	Middle shelf (50–100 m)
	*Lenticulina hughesi*	Oxic	Epifaunal				Calc.		
	*Bolivina saidi*	Dysoxic	Infaunal				Calc.		
	*Gyroidina *sp1	Suboxic	Epifaunal				Calc.		
	*Gyroidina *sp2.	Suboxic	Epifaunal				Calc.		
6	*Haplophragmides *sp	Oxic/dysoxic	Epifaunal/shallow infaunal	1.6 to 2.4	0.13 to 0.2	53 to 83	Aggl.	Low oxic	Middle shelf (50–100 m)
	*Lagena apiopleura*	Suboxic	Shallow infaunal				Calc.		
7	*Cyclammina incisa*	Suboxic	Infaunal	1.6 to 3.1	0.12 to 0.16	70 to 75	Calc.	Suboxic to oxic	Middle shelf (50–100 m)
	*Baggina regularis*	Suboxic	Infaunal				Calc.		
	*Eponoides repandus*	Oxic	Epifaunal				Calc.		
8	*Bolivina pseudospissa*	Dysoxic	Infaunal	0.7 to 1.6	0.16 to 0.3	68 to 71	Calc.	Dysoxic	Outer shelf to upper slope (<200 m)
	*Uvigerina subperegrina*	Dysoxic	Infaunal				Calc.		
9	*Bolivina brevior*	Dysoxic	Infaunal	0.1 to 1.9	0.09 to 0.1	10 to 20	Calc.	Dysoxic to oxic	Inner shelf (0–50 m)
	*Globocassidulina monicana*	Dysoxic	infaunal/epifaunal				Calc.		
	*Cibicides ellisi ellisi*	Oxic	Epifaunal				Calc.		
10	*Bolivina fastigia*	Dysoxic	Infaunal	2.4 to 3.2	0.18 to 0.2	58 to 75	Calc.	Dysoxic	Middle to outer shelf (50–150 m)
	*Cassidulina cruysi*	Dysoxic	Infaunal/epifaunal				Calc.		
	*Uvigerina barbatula*	Dysoxic	Infaunal				Calc.		

##### Biofacies 1 (*Buliminella*–*Uvigerina* biofacies)

The *Buliminella–Uvigerina* biofacies is only recognized in ARS-6 borehole in the Lower Rudeis Formation. It is dominated by shales with minor intercalations of limestones. This biofacies is characterized by a high abundance of *Buliminella elegantissima*, *Buliminella subfusiformis*, *Uvigerina senticosa*, *Uvigerina venusta* (Fig. 9; Table 3). It is dominated by epifaunal, infaunal deep fauna including *Lenticulina smileyi*, *Chilostomella ovoidea* and agglutinated species *Bathysiphon taurinensis.* The genus *Uvigerina* suggests outer shelf to bathyal settings (< 200 m)^114^. The genus *Chilostomella* is more abundant in upper slope setting of 200 to 500 m water depth^81^^96^,). The *Lenticulina* is regarded as representative of middle shelf to upper slope faunas, whereas the genus *Buliminella* suggests temperate shelf to upper slope settings^115^. The high relative abundances of *Buliminella*, *Uvigerina*, *Lenticulina* and *Chilostomella* indicate outer shelf to upper slope settings. This biofacies is characterized by a high planktonic/benthic (P/B) ratio (50–80), elevated agglutinated/calcareous ratio (4.5–13.6), and a high infaunal/epifaunal (I/E) ratio (53–78). It also exhibits moderate dominance (0.11–0.15), high species diversity (Fisher’s α: 2.04–3.9), and high gamma-ray values (22–103 API units) (Fig. 9).

##### Biofacies 2 (*Bolivina*–*Nonion* biofacies)

This biofacies is recorded exclusively in ARS-6 borehole within the Nukhul Formation. It is dominated by shale with thin limestone beds and increasingly frequent sandstone intercalations, particularly toward the top of the formation. The assemblage is characterized by the presence of *Bolivina dilatata*, *Bolivina suberpa*, *Nonion scapha*, *Pseudononion basispinata* and *Cibicides dutemplei* (Fig. 9; Table 3). The genus *Cibicides* is associated with depths extending from shelf to upper slope environments^115–117^. The genus *Nonion* occurs in inner to middle shelf environments corresponding to paleodepths less than 180 m^115,118^. This biofacies indicates deposition in an inner shelf environment. It is dominated by a low planktonic/benthic (P/B) ratio (10–25), a zero agglutinated/calcareous ratio, and a low infaunal/epifaunal (I/E) ratio (10–38). Additionally, it exhibits low dominance (0.10–0.12), low species diversity (Fisher’s α: 0.1–2.0), and low gamma-ray values (9–77 API units) (Fig. 9).

##### Biofacies 3 (*Cibicides*–*Epistominella* biofacies)

The *Cibicides–Epistominella *biofacies is identified exclusively in the SIDRI-20 borehole within the Kareem Formation. It is dominated by barren anhydrite facies that grade upward into gray fossiliferous shale with thin sandstone streaks intercalated with anhydrite. This biofacies is characterized by the presence of *Quiquiloculina seminula*, *Spiroloculina tenuis*, *Cibicidoides praecinctus* and *Epistominella smithi* (Fig. 10; Table 3). The occurrence of *Quinqueloculina* suggests deposition in marine-hypersaline, ranging from shelf to bathyal settings^115,119^^**,**^^120^. In contrast, the genus *Epistominella* inhabits inner shelf to upper slope areas^81^^;^^96^^,^^115^. This biofacies is characterized by a low planktonic/benthic (P/B) ratio (10–15), very low species diversity (Fisher’s α: 0.2–1.5), low dominance (0.09–0.10), and low gamma-ray values (53–63 API units) (Fig. 10). The abundance of shallow-water fauna, combined with barren anhydrite intervals at the base of the Kareem Formation, suggests deposition within a restricted marine lagoonal setting.

##### Biofacies 4 (*Nodosaria*–*Nonionella* biofacies)

The *Nodosaria–Nonionella* biofacies is exclusively reported in the SIDRI-20 borehole, occurring at the top of the Nukhul Formation and extending into the base of the Lower Rudeis Formation. It is composed predominantly of shale deposits. This biofacies is dominated by *Nonionella miocenica*, *Nodosaria ovicula*, *Bolivina conica*, *Loxostomoides digitata* and agglutinated species of *Ammobaculites sp.*, suggesting deposition in inner to middle shelf environments (^121–125^; Fig. 10; Table 3). The presence of inner to middle shelf species such as *Nonionella* suggests low-oxygen conditions^115^. It is dominated by elevated values of the planktonic/benthic (P/B) ratio (46–72), agglutinated/calcareous ratio (3.3–11.1), and infaunal/epifaunal (I/E) ratio (40–60). This biofacies is further characterized by higher dominance (0.11–0.20), high species diversity (Fisher’s α: 1.5–3.4), and high gamma-ray values (40–107 API units) (Fig. 10).

##### Biofacies 5 (*Lenticulina*–*Gyroidina* biofacies)

The *Lenticulina–Gyroidina* biofacies is recorded exclusively in the ARS-6 borehole, within the middle part of the Lower Rudeis Formation (Fig. 9). It is composed of shale with intercalated sandstone and limestone beds. This biofacies is marked by a higher abundance of deep-water taxa, including *Lenticulina budensis*, *Lenticulina hughesi*, *Bolivina saidi* and *Gyroidina* spp. (Table 3). The genera *Lenticulina* and *Gyroidina* are considered indicative of middle shelf settings^96,116,126^. It is dominated by a high planktonic/benthic (P/B) ratio (60–80) and an elevated infaunal/epifaunal (I/E) ratio (16–38). In addition, it is marked by moderate dominance (0.11–0.14), high species diversity (Fisher’s α: 2.9–3.4), and low gamma-ray values (15–62 API units) (Fig. 9).

##### Biofacies 6 (*Haplophragmides* biofacies)

The *Haplophragmides* biofacies is recorded at the top of the Lower Rudeis Formation in the ARS-6 borehole and becomes more common within the Lower Rudeis Formation in the SIDRI-20 borehole (Figs. 9, 10). It is composed of shale with intercalated sandstone and limestone beds. This biofacies is distinguished by the high abundance of two species: *Haplophragmides* sp. and *Lagena apiopleura* (Table 3). *Haplophragmides* species are typically dominant in inner to middle shelf sandy facies environments (^115,118,121–125^). It is dominated by a high planktonic/benthic (P/B) ratio (53–83), elevated dominance (0.13–0.20), high species diversity (Fisher’s α: 1.6–2.4), and low gamma-ray values (40–86 API units) (Figs. 9, 10).

##### Biofacies 7 (*Eponoides* biofacies)

The *Eponides* biofacies is reported exclusively in the ARS-6 borehole within the Lower Rudeis Formation. It is composed of shale with intercalated sandstone and limestone beds. This biofacies is characterized by a higher abundance of *Cyclammina incisa*, *Baggina regularis*, and *Eponides repandus* (Fig. 9; Table 3). *Eponides* species are typically associated with inner to middle shelf environments (^115,118,121–125^). It is dominated by a high planktonic/benthic (P/B) ratio (70–75). This biofacies is further characterized by moderate Dominance (0.12–0.16), high species diversity (Fisher’s α: 1.6–3.1), and low gamma-ray values (31–63 API units) (Fig. 9).

##### Biofacies 8 (*Bolvina*–*Uvigerina* biofacies)

This biofacies is reported exclusively in the ARS-6 borehole within the Lower Rudeis Formation. It is composed mainly of shale with minor sandstone and limestone streaks. The assemblage is characterized by a high abundance of *Uvigerina subperegrina* and *Bolivina pseudospissa* (Fig. 9; Table 3). The genus *Uvigerina* is considered indicative of maximum flooding surfaces^95^ and typically occurs in outer neritic to bathyal settings^114^. This biofacies also exhibits a high planktonic/benthic (P/B) ratio (68–71), elevated dominance (0.16–0.30), moderate species diversity (Fisher’s α: 0.7–1.6), and low gamma-ray values (29–75 API units) (Fig. 9).

##### Biofacies 9 (*Bolivina*–*Cibicides* biofacies)

The *Bolivina–Cibicides* biofacies is documented exclusively in the ARS-6 borehole, where it occurs within the Nukhul, Lower Rudeis, and Kareem formations. It is dominated by shale with thin intercalations of limestone and sandstone. This biofacies is characterized by a higher abundance of three species: *Bolivina brevior*, *Globocassidulina monicana*, and *Cibicides ellisi ellisi* (Fig. 9; Table 3). The co-occurrence of *Bolivina* and *Cibicides* suggests deposition in inner shelf environments^81,96,115^. Quantitatively, it is marked by a low planktonic/benthic (P/B) ratio (10–20), low dominance (0.09–0.10), moderate species diversity (Fisher’s α: 0.1–1.9), and low gamma-ray values (13–80 API units) (Fig. 9).

##### Biofacies 10 (*Uvigerina*–*Cassidulina* biofacies)

The *Uvigerina* biofacies is recorded in the ARS-6 and SIDRI-20 boreholes within the Lower Rudeis Formation. It is dominated mainly by shale with minor limestone and sandstone streaks. This biofacies is characterized by a high abundance of *Uvigerina barbatula* (Figs. 9, 10; Table 3), along with subordinate occurrences of *Bolivina fastigia* and *Cassidulina cruysi*. These taxa are indicative of middle shelf to outer neritic environments. The genus *Uvigerina* is typically associated with outer neritic to bathyal settings^114^ and is known to mark maximum flooding surfaces^95^. This biofacies also exhibits a high planktonic/benthic (P/B) ratio (58–75), elevated dominance (0.18–0.20), high species diversity (Fisher’s α: 2.4–3.2), and low gamma-ray values (16–76 API units) (Figs. 9, 10).

## Discussion

### Depositional model

**Fig. 13:**
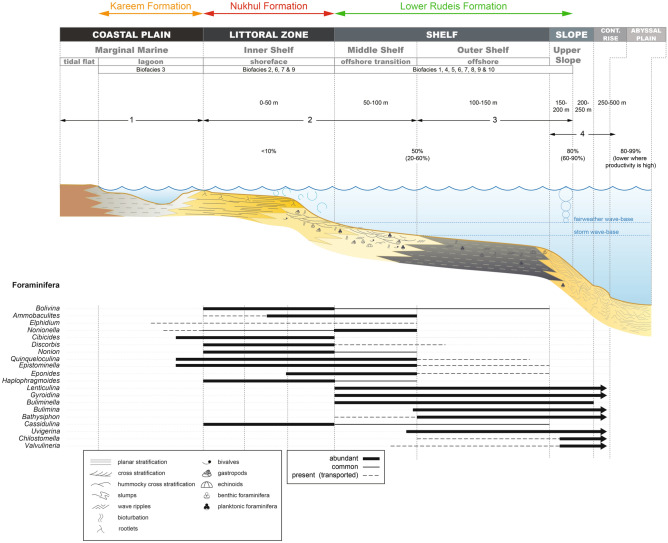
Depositional model for the Lower–Middle Miocene succession in the Abu Rudeis–Sidri Field, showing interpreted depositional environments, distribution of benthic foraminiferal biofacies, and representative benthic taxa across a generalized shelf-to-slope transect. The model illustrates three depositional phases: (1) inner shelf (Nukhul Formation), (2) middle to upper shelf/slope (Lower Rudeis Formation), and (3) restricted lagoon (Kareem Formation), placed within a paleobathymetric framework. Foraminiferal depth ranges are based on^81,96^ and^115^.

The depositional model is based on benthic foraminiferal assemblages and lithofacies integrated with wireline log data. Vertical changes in biofacies, gamma-ray profiles, and lithologic patterns are used to reconstruct shifts in depositional environments. The Lower–Middle Miocene succession reflects three depositional phases during the evolution of a siliciclastic-dominated shelf-to-slope system, driven by tectonic activity and relative sea-level fluctuations (Fig. 13).

Depositional Phase 1: Burdigalian – inner shelf.

The continental deposition of the Abu Zenima Formation was terminated by a marine transgression, leading to the establishment of shallow subtidal marine conditions during the deposition of the Nukhul Formation. This occurred during early rift extension and was accompanied by increasing subsidence. The Nukhul Formation comprises shales interbedded with thin limestone and sandstone beds, and is characterized by *Bolivina–Nonion* and *Bolivina–Cibicides* biofacies (biofacies 2 and 9), dominated by *Bolivina* spp, *Nonion* sp*.*, *Eponoides* sp. and *Cibicides* spp. These assemblages are indicative of oxygenated, shallow subtidal inner-shelf environments (~ 30–50 m depth).

The lower Nukhul interval is marked by high abundances of *Nonion* sp. (15–20%), *Bolivina* spp. (23–50%), *Cibicides* sp. (19–30%), and *Eponides* sp. (31–61%) (Figs. 11, 12), alongside a general upward decrease in gamma-ray readings from 9 to 80. In contrast, the upper interval shows a marked decrease in *Nonion* sp. and *Eponides* sp., together with declining gamma-ray values, suggesting a shallowing-upward trend and progradation. The low planktonic/benthic (P/B) ratio, low total foraminiferal number (TFN), and low species diversity support deposition on a shallow, low-energy inner shelf (Fig. 13).

Depositional Phase 2: Burdigalian – middle shelf to upper slope.

A major marine flooding surface caps the Nukhul Formation, separating it from the overlying Lower Rudeis Formation in the Abu Rudeis–Sidri Field, east-central Gulf of Suez (Figs. 5–8). Deposited during the rift-climax phase under rapid tectonic subsidence and fault-block rotation, the Lower Rudeis Formation comprises gray to greenish-gray, highly argillaceous, calcareous shales interbedded with off-white to yellow sandstones, siltstones, and thin limestones. This succession reflects an overall transgressive trend from shallow to deeper subtidal environments, with elevated gamma-ray values from 22 to 107 in the lower part that gradually decline upward, suggesting increasing accommodation and varying sediment input.

The Lower Rudeis Formation contains a diverse array of benthic foraminiferal biofacies (Biofacies 1, 4–10), including *Buliminella–Uvigerina*, *Lenticulina–Gyroidina*, and *Uvigerina* assemblages (Figs. 11, 12). The lower interval is dominated by deep-water taxa such as *Buliminella* spp., *Uvigerina* sp. (< 200 m^114^), *Lenticulina* sp., *Chilostomella* sp. (200–500 m; 81; 96), and *Gyroidina* spp. (upper slope^116,126^), alongside agglutinated forms like *Bathysiphon* sp. and *Ammobaculites* sp. These assemblages, along with high gamma-ray values, indicate deposition in outer shelf to upper slope environments during peak tectonic subsidence.

Upsection, the biofacies shift to mixed assemblages in which deep-water taxa (*Uvigerina*, *Chilostomella*, *Gyroidina*) co-occur with shallow forms such as *Eponides*, *Bolivina*, and *Cibicides*, suggesting a gradual shoaling to middle–outer shelf settings. This is consistent with declining gamma-ray values (Fig. 9–12).

These trends are further supported by high planktonic/benthic (P/B) ratios (46–83), elevated total foraminiferal numbers (30–50 individuals/g), moderate to high species diversity (Fisher’s α: 2.4–3.4), and dominance values up to 0.20 (Figs. 9, 10). Together with the integrated gamma-ray profiles, these features reflect deposition across a bathymetric gradient from middle shelf to upper slope (~ 50–200 m), with a general shallowing-upward trajectory linked to waning tectonic subsidence (Fig. 13).

Depositional Phase 3: Langhian – restricted marine lagoon.

The Kareem Formation records a regressive transition from open-marine to restricted lagoonal conditions. The basal interval consists of barren, white to milky anhydrite, interpreted as sabkha or hypersaline lagoon deposits, characterized by low gamma-ray and high resistivity values^42^^**;**^^127^. Up section, the anhydrite grades into gray fossiliferous shales interbedded with thin sandstone and residual anhydrite beds, exhibiting relatively low gamma-ray and resistivity signatures. These shales contain the shallow-water *Cibicides–Epistominella* biofacies (Biofacies 3), dominated by *Quinqueloculina*, *Cibicides*, and *Epistominella* (Figs. 11, 12). These taxa are associated with inner to middle shelf settings, and their co-occurrence with evaporites and low-diversity assemblages suggests a restricted, low-energy lagoonal environment^81^^**;**^^96^^**;**^^115^. Further support comes from the low planktonic/benthic ratio, low total foraminiferal number (TFN), and minimal species richness. Together with the evaporitic lithofacies, these features indicate deposition in a shallow (0–5 m), oxygen-stressed, hypersaline setting during relative sea-level fall and post-climax tectonic uplift (Fig. 13).

### Sequence stratigraphy

**Fig. 14:**
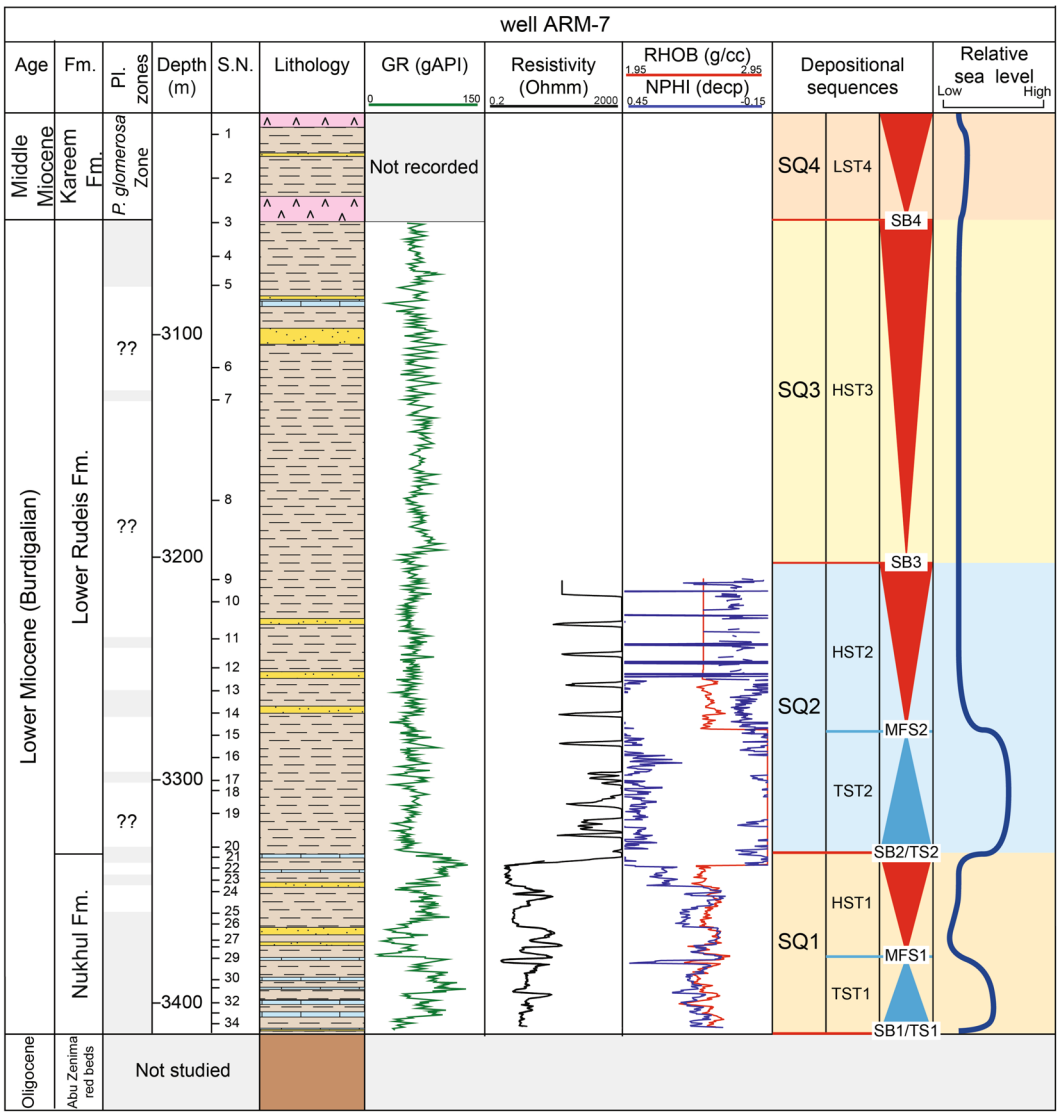
Sequence stratigraphic framework in well ARM-7.

**Fig. 15:**
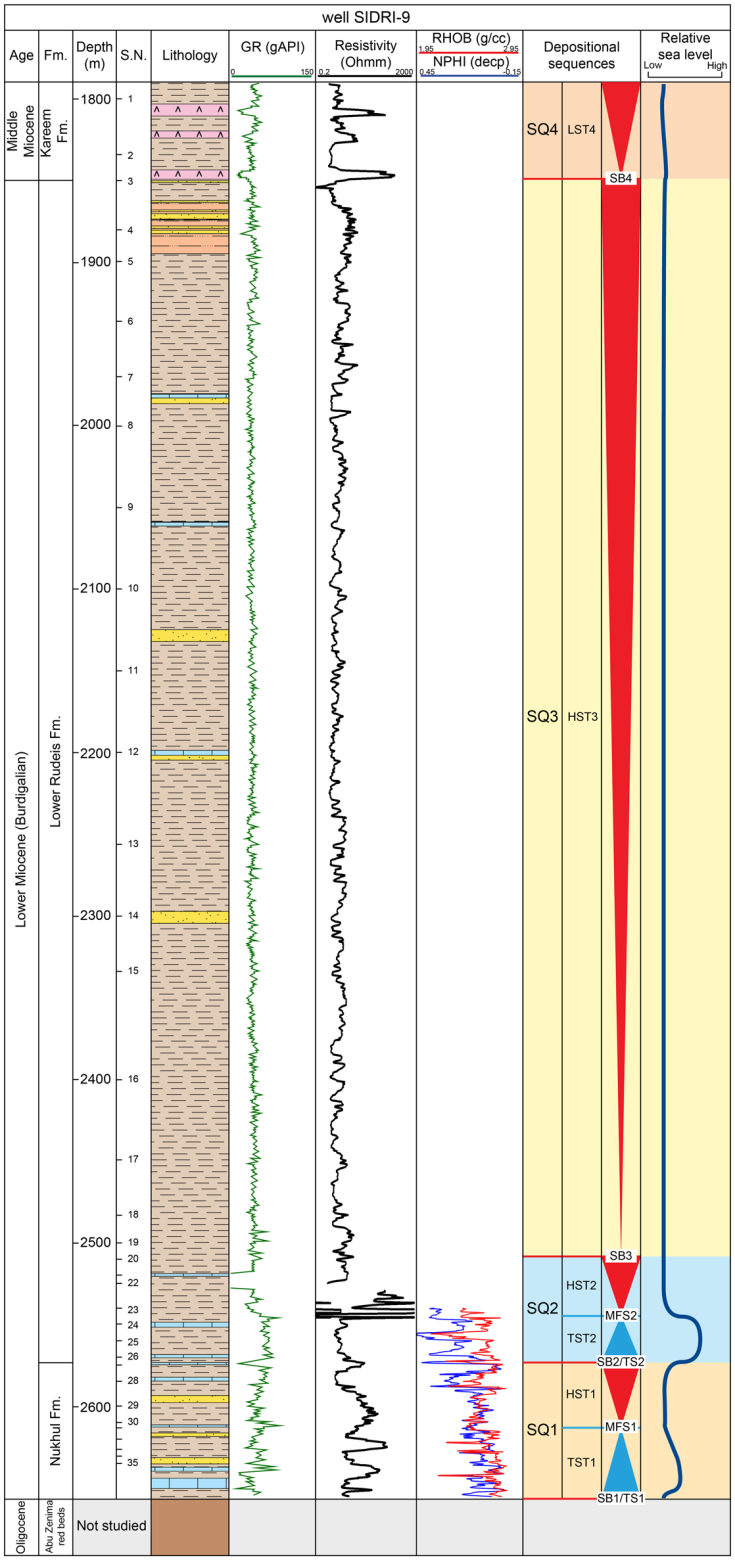
Sequence stratigraphic framework in well SIDRI-9.

**Fig. 16:**
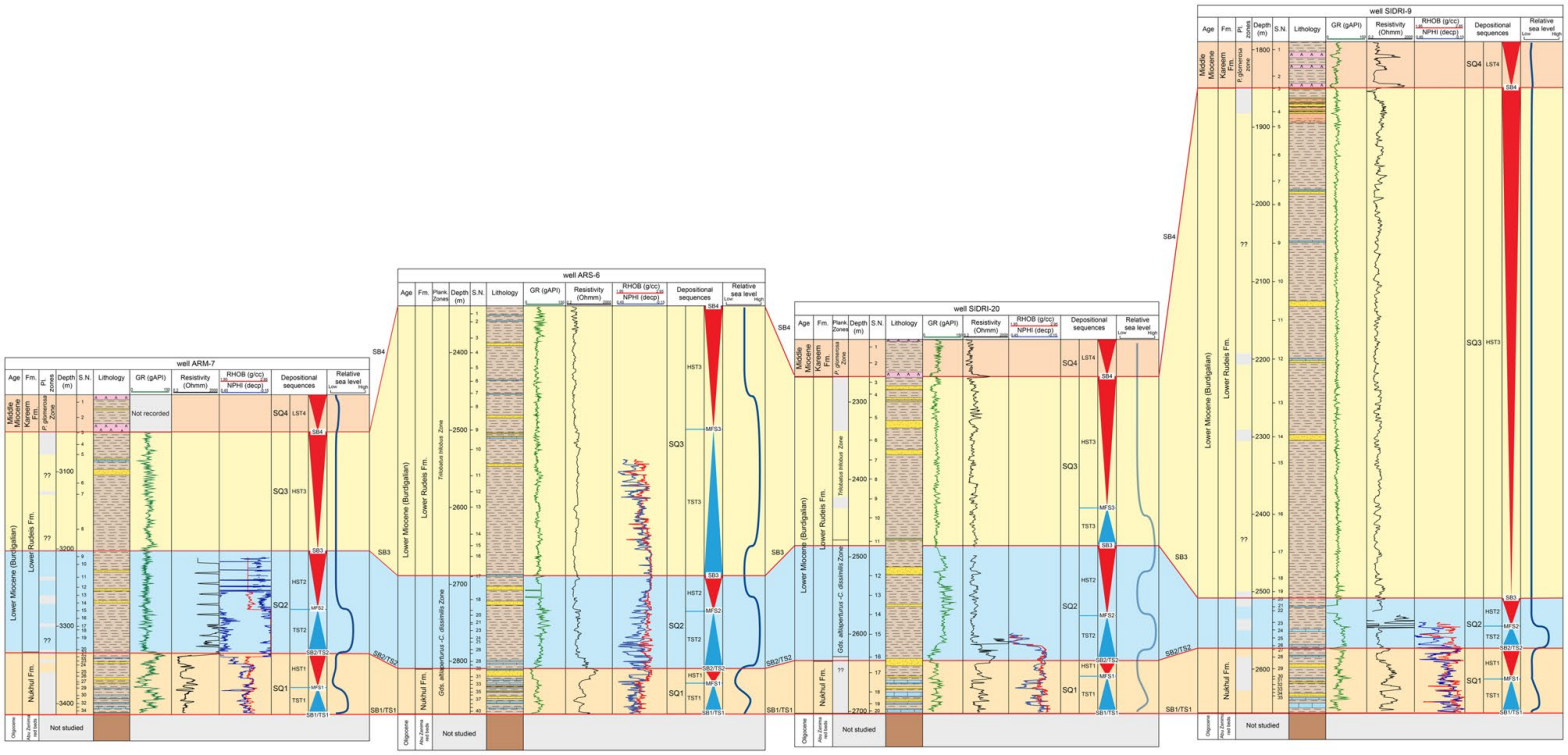
Sequence stratigraphic correlation along the cross-section shown in Fig. 1C, illustrating thickness variations in Burdigalian–Langhian sequences across the study area.

This study develops a sequence stratigraphic framework for the early syn-rift Miocene succession in the Abu Rudeis–Sidri Field, east-central Gulf of Suez, based on the integration of wireline logs, benthic foraminiferal biofacies, gamma-ray patterns, and seismic interpretation from four onshore wells (ARM-7, ARS-6, SIDRI-20, and SIDRI-9; Fig 9, Fig 10, Fig 11, Fig 12, Fig 14, Fig 15, Fig 16). Although seismic imaging in the study area is generally poor, primarily due to Miocene evaporite facies that obscure reflection continuity, this limitation is mitigated by calibrating seismic picks with well-log signatures and biofacies trends. This correlation allows for higher-resolution subsurface interpretation crucial for evaluating stratigraphic architecture and reservoir heterogeneity.

The available 2D seismic data were reinterpreted along 19 profiles transecting the studied wells to establish the structural and stratigraphic framework of the Miocene succession. The seismic sections reveal a series of tilted fault blocks, typical of the half-graben architecture of the central Gulf of Suez. Syn-rift reflectors display wedge-shaped geometries thinning toward the master faults, whereas post-rift reflectors onlap the tilted blocks. Key seismic horizons were correlated with well data using synthetic seismograms generated from the density and sonic logs of wells ARM-7 and ARS-6. This well-to-seismic tie ensures that major stratigraphic surfaces—namely, sequence boundaries (SB1–SB4) and maximum flooding surfaces (MFS1–MFS3)—are accurately positioned within the seismic framework. Reflections corresponding to these surfaces exhibit distinct terminations (onlap, toplap, and truncation), consistent with the sequence stratigraphic interpretation derived from wireline and biostratigraphic data.

Seismic reflections also reveal lateral variations in thickness and internal architecture within the Nukhul, Rudeis, and Kareem formations. These variations reflect differential subsidence across adjacent fault blocks, providing evidence for strong tectonic control on accommodation during the rift-climax stage. The geometry and continuity of the reflectors indicate that the studied field lies near the boundary between two major half-graben systems and contains localized depocenters along NE–SW-trending faults. Although detailed time- and depth-structure maps were withheld due to data-use restrictions, the representative seismic profiles adequately illustrate the stratigraphic architecture and inter-well relationships. These profiles demonstrate how seismic observations complement high-resolution biostratigraphic and log-based analyses, reinforcing the interpretation of depositional sequences in the Abu Rudeis–Sidri area.

The framework, following^85^and the tripartite systems tract model of^128^, identifying four third-order depositional sequences (SQ1–SQ4) bounded by sequence boundaries (SB1–SB4) and maximum flooding surfaces (MFS1–MFS3). These are primarily developed within the Lower Miocene Nukhul and Rudeis formations and the basal Kareem Formation. Stratigraphic surfaces are defined based on stacking patterns, abrupt gamma-ray shifts, and transitions in benthic foraminiferal assemblages. The basal transgression (SB1/FS1) marks a shift from non-marine to inner-shelf faunas (e.g., *Bolivina*, *Nonion*, *Cibicides*), while SB2/FS2 corresponds to an abrupt deepening into outer shelf–upper slope assemblages (*Buliminella*, *Uvigerina*, *Lenticulina*). A gradual return to shallower and restricted faunas (*Eponides*, *Gyroidina*, *Cibicides*, then *Epistominella*, *Quinqueloculina*) records a shoaling trend associated with waning subsidence and tectonic uplift. The absence of *Globigerinoides primordius* at the Chattian–Burdigalian boundary (SB1) supports the interpretation of a regional tectonic hiatus. The following subsections present the stratigraphic surfaces and systems tracts that define each depositional sequence.

#### Stratigraphic surfaces

The stratigraphic surfaces denote changes in stratal stacking patterns and include sequence boundary (SB), transgressive surface (TS), and maximum flooding surface (MFS). The surfaces are identified based on biostratigraphic break, erosional surfaces, subaerial exposures, and abrupt gamma-ray log break^128,129^.

##### Sequence boundaries

Four third-order sequence boundaries (SB1–SB4) are recognized in the Abu Rudeis–Sidri succession, each representing an erosional unconformity or stratigraphic hiatus. Two of these boundaries (SB1 and SB2) are compound surfaces, modified by subsequent marine transgressions and interpreted as both sequence boundaries and flooding surfaces (SB1/FS1 and SB2/FS2). These surfaces are defined based on abrupt shifts in facies, gamma-ray trends, and benthic foraminiferal biofacies (Fig 11, Fig 12, Fig 14, Fig 15, Fig 16).

###### SB1 – base nukhul unconformity/TS1 composite surface

SB1 marks the erosional contact between the Abu Zenima and Nukhul formations. This surface is associated with a sharp facies transition from nonmarine continental red beds to shallow-marine inner shelf deposits, representing a major tectono-eustatic hiatus of Aquitanian age. It is modified by a transgressive flooding event, marked by the LO of *Globigerinoides altiaperturus*(~ 20.5 Ma^103^;), and corresponds to TS1. The overlying transgressive systems tract (TST1) is supported by an upward increase in gamma-ray values and the appearance of inner shelf benthic biofacies (Biofacies 2 and 9), dominated by *Bolivina*, *Nonion*, and *Cibicides*.

###### SB2 – top nukhul unconformity/TS2 composite surface

SB2 marks the erosional boundary between the Nukhul and Lower Rudeis formations and reflects a regional unconformity associated with intensified extensional faulting and rapid tectonic subsidence during the rift climax phase. The surface is overlain by a major marine transgression (TS2), corresponding to the T10 flooding event (~ 19.7 Ma^54^;). It is defined by a sharp facies change from shallow inner-shelf deposits to deeper marine calcareous shales, a pronounced increase in gamma-ray values, and the abrupt appearance of deep-water benthic foraminiferal assemblages (*Buliminella*, *Uvigerina*, *Lenticulina*; Biofacies 1 and 4). These changes indicate a rapid shift into outer shelf to upper slope settings, driven by increased accommodation and relative sea-level rise. The SB2–TS2 composite surface represents a combined unconformity and marine flooding surface, consistent with tectono-eustatic controls on sequence development in the Gulf of Suez (Fig 11, Fig 12, Fig 14, Fig 15, Fig 16).

###### SB3 – intra-lower rudeis unconformity

SB3 marks a subtle stratigraphic break within the Lower Rudeis Formation and defines the boundary between depositional sequences SQ2 and SQ3. It corresponds to the regional T20 surface of^54^, dated at approximately 17.6 Ma (HO of *Catapsydrax dissimilis*), and is interpreted as a minor unconformity linked to tectonic reorganization and localized uplift during the late Burdigalian.

This surface is recognized by a modest change in gamma-ray stacking pattern, transitioning from retrogradational to aggradational motifs, and is accompanied by a turnover in benthic foraminiferal assemblages. Deep-water taxa such as *Buliminella*, *Uvigerina*, and *Chilostomella* below the surface are replaced by mixed-depth assemblages including *Eponides*, *Gyroidina*, and *Cibicides* (Biofacies 6–9), indicating relative shoaling and reduced subsidence. This transition is clearest in wells SIDRI-20 and ARS-6 (Fig 11, Fig 12, Fig 14, Fig 15, Fig 16), but more difficult to trace in ARM-7 and SIDRI-9 due to lower faunal recovery and subdued log signatures.

###### SB4 – base kareem unconformity

SB4 represents a major erosional unconformity separating the marine shales of the Lower Rudeis Formation from the anhydrite-rich, restricted lagoonal deposits of the Kareem Formation in the Abu Rudeis–Sidri Field (Fig 11, Fig 12, Fig 14, Fig 15, Fig 16). This boundary, dated near the Burdigalian–Langhian boundary (~ 15.99 Ma^130^), is marked by a sharp drop in gamma-ray values, increased resistivity, and the disappearance of planktonic foraminifera, reflecting a relative sea-level fall coupled with tectonic uplift. Above SB4, the appearance of Biofacies 3 (*Cibicides*–*Epistominella*) indicates restricted inner shelf to lagoonal conditions (~ 0–5 m depth), contrasting with the deeper-water assemblages (e.g., *Buliminella*, *Uvigerina*) of the underlying Rudeis Formation. These sharp faunal and facies shift supports the interpretation of progressive restriction during the late syn-rift phase.

#### Depositional sequences (SQ1-SQ4)

This section delineates four third-order depositional sequences (SQ1–SQ4) based on gamma-ray stacking patterns, lithofacies trends, and benthic foraminiferal biofacies calibrated to paleobathymetric ranges. Each sequence is subdivided into systems tracts and bounded by regionally correlatable stratigraphic surfaces.

##### Depositional sequence SQ1 (early burdigalian – nukhul formation)

**Fig. 17:**
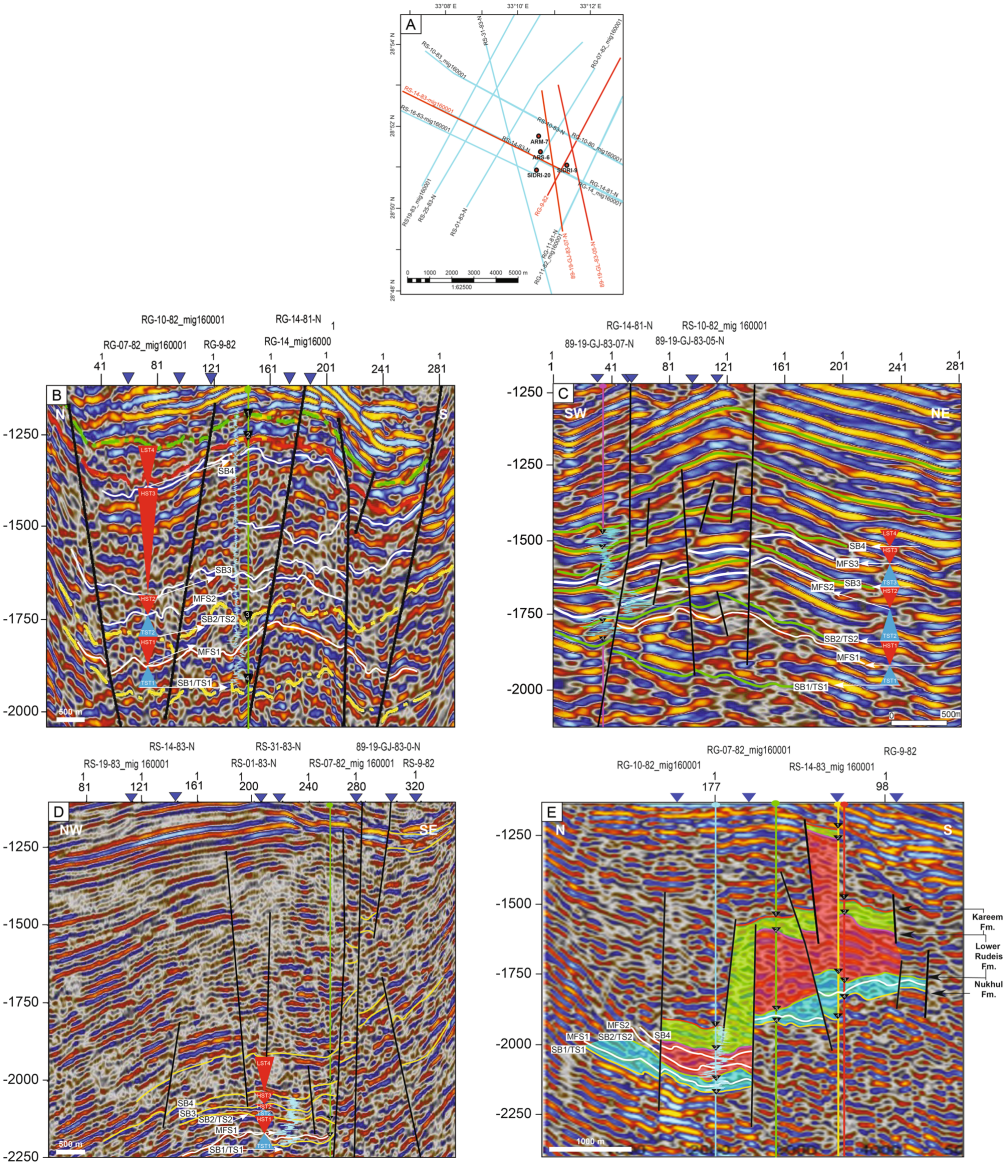
Interpreted seismic cross-sections illustrating the structural framework and sequence architecture across the Abu Rudeis–Sidri Field: (**A**) Seismic line locations. (**B**) Line 98–19-GJ-83–07-N (N–S). (**C**) Line RG-9–82 (SW–NE). (**D**) Line 89–19-GL (NW–SE). (**E**) Line RS-14-83_mig160001 (N–S). These sections highlight syn-rift fault geometries, sequence boundaries, and accommodation patterns shaped by tectonic controls.

Depositional Sequence 1 (SQ1) corresponds to the shallow marine deposits of the Nukhul Formation. It is bounded below by a composite surface comprising an erosional unconformity and a transgressive surface (SB1/TS1), marked by the LO of *Globigerinoides altiaperturus* (~ 20.5 Ma^103^), and above by another composite surface (SB2/TS2), correlated with the T10 event (~ 19.7 Ma^54^). This third-order sequence spans approximately 0.7 Ma and represents a transgressive–regressive cycle tied to the initial marine flooding of the Gulf of Suez rift, transitioning from non-marine continental red beds to inner shelf marine conditions featuring inner shelf biofacies. On seismic profiles, SQ1 is characterized by low-continuity, sub-parallel reflections (Fig. 17). No lowstand systems tract (LST) is recorded, likely due to rapid tectonic subsidence during early rifting. The sequence includes transgressive (TST1), maximum flooding (MFS1 within TST1 and HST1), and highstand (HST1) systems tracts.

###### Transgressive Systems Tract (TST1)

TST1 is developed across all four studied wells, with a thickness of 33–50 m. It consists of interbedded shale, sandstone, and limestone and exhibits a bell-shaped gamma-ray trend and moderate to low resistivity (Figs. 9–12). In well ARS-6, it is characterized by Biofacies 2 and 9, dominated by *Bolivina spp.* (23–50%), *Nonion sp.* (15–20%), and *Cibicides sp.* (19–30%), with minor *Eponides* and *Valvulineria*. These assemblages indicate deposition on a shallow inner shelf (estimated depths of 20–50 m; Sections "Biofacies 2 (Bolivina–Nonion biofacies)" and "Biofacies 9 (Bolivina–Cibicides biofacies)"). Moderate planktonic/benthic (P/B) ratios (20–25) and low total foraminiferal numbers (TFN: 13–16 individuals/g) suggest restricted marine conditions with limited oceanic circulation. Although biostratigraphic resolution is limited in SIDRI-20 due to barren intervals, correlation with ARS-6 is supported by consistent gamma-ray profiles.

###### Maximum flooding surface (MFS1)

MFS1 is placed within the upper part of the Nukhul Formation, dated to approximately 20.0 Ma in well ARS-6. It is identified by peak gamma-ray values and the highest diversity and abundance of inner shelf foraminifera, notably *Eponides repandus*, *Bolivina*, and *Cibicides* (Biofacies 2). This surface reflects maximum accommodation and marine transgression under continued tectonic subsidence.

###### Highstand systems tract (HST1)

The HST1 occurs above MFS1, ranging in thickness from 25 m (SIDRI-20) to 45 m (ARM-7). It is composed of sandstone, shale, and limestone and displays a funnel-shaped gamma-ray trend (Fig 9, Fig 10, Fig 14, Fig 15, Fig 16). Biofacies 2 remains dominant in ARS-6, with *Bolivina spp.* (23–33%) and *Cibicides sp.* (19–33%), indicative of continued inner shelf deposition (~ 20–50 m; Section "Biofacies 2 (Bolivina–Nonion biofacies)"). Slightly higher P/B ratios (38–40) and comparable TFN (13–15 individuals/g) suggest minor deepening near the flooding peak, followed by a regressive shift. The subtle progradational stacking reflects sediment infill under gradually declining accommodation space.

##### Depositional sequence SQ2 (19.7–17.62 Ma; lower rudeis formation)

Depositional Sequence 2 (SQ2) corresponds to the lower part of the Lower Rudeis Formation and spans approximately 2.08 Ma. It is bounded below by the composite surface SB2/TS2 (~ 19.7 Ma; T10 event^54^) and above by the sequence boundary SB3, marked by the HO of *Catapsydrax dissimilis* (~ 17.62 Ma^103^). SQ2 records a pronounced marine transgression and subsequent highstand during the early Burdigalian and is well expressed in wells ARS-6 and SIDRI-20. Seismic profiles show low-continuity, sub-parallel reflectors (Fig. 17). Due to rapid tectonic subsidence and limited sediment supply, no lowstand systems tract is recognized. The sequence comprises transgressive (TST2), maximum flooding (MFS2), and highstand (HST2) systems tracts.

###### Transgressive systems tract (TST2)

TST2 lies at the base of the Lower Rudeis Formation and varies from 35 m (SIDRI-9) to 76 m (ARS-6) in thickness. It is composed mainly of shale with minor limestone interbeds and shows a general increase in gamma-ray values (Figs. 9–12). In ARS-6 and SIDRI-20, this tract contains Biofacies 1 and 4, dominated by *Buliminella spp.* (15–42%), *Bulimina sp.* (8–38%), *Uvigerina sp.* (15–38%), *Lenticulina sp.* (15–45%), and agglutinated taxa such as *Bathysiphon* and *Ammobaculites*. These assemblages indicate deposition on the outer shelf to upper slope (paleodepths > 150 m; Sections "Biofacies 1 (Buliminella–Uvigerina biofacies)" and "Biofacies 4 (Nodosaria–Nonionella biofacies)"). High P/B ratios (46–80), moderate calcareous/agglutinated ratios (5–16%), and elevated TFN (30 individuals/g) support rapid marine deepening during peak subsidence.

###### Maximum flooding surface (MFS2)

MFS2 is identified near the top of TST2 at ~ 19.0 Ma and corresponds to the maximum gamma-ray peaks and the highest diversity of deep-water benthic foraminifera *Uvigerina*, *Buliminella*, *Lenticulina* (Biofacies 1 and 4) in wells ARS-6 and SIDRI-20 (Figs. 11–12). These biofacies reflect peak marine transgression and the maximum accommodation space generated by rapid tectonic subsidence during the rift climax phase.

###### Highstand systems tract (HST2)

The HST2 occurs above MFS2 and occupies the middle part of the Lower Rudeis Formation, ranging in thickness from 30 m (SIDRI-9) to 95 m (ARS-6) (Figs. 9, 10, 14–16). It is composed of shales with intercalated fine-grained sandstones and limestones, contrasting with the underlying shale-dominated TST2, and exhibits a gradual decrease in gamma-ray values, producing a blocky-to-funnel-shaped log motif. This lithologic and log character reflects a shift to shallower depositional settings, supported by progradational stacking.

In ARS-6, HST2 contains Biofacies 5, dominated by *Bolivina* spp. (12–29%), *Cibicides* spp. (11–46%), *Epistominella* sp. (5–10%), *Lenticulina* sp. (14–35%), *Uvigerina* spp. (0–10%), and *Gyroidina* sp. (21–27%), indicative of middle to inner shelf deposition (~ 50–100 m). In SIDRI-20, Biofacies 6 prevails, with *Haplophragmoides* sp. (10–15%) and *Lagena* sp. (9–12%), suggesting shallower inner shelf to restricted conditions (< 50 m; Section "Biofacies 6 (Haplophragmides biofacies)"). Moderate to high P/B ratios (50–83%) and TFN values (16–20 individuals/g) indicate a regressive trend into more restricted marine environments. In ARM-7 and SIDRI-9, the HST2 is identified based on similar gamma-ray trends and stratigraphic correlation, despite limited biostratigraphic control.

##### Depositional sequence SQ3 (17.62–16.9 Ma; upper lower rudeis formation)

Depositional Sequence 3 (SQ3) comprises the upper Lower Rudeis Formation within the *Trilobatus trilobus* Zone (~ 17.62–16.9 Ma) in ARS-6 and SIDRI-20 (Fig 9, Fig 10, Fig 14, Fig 15, Fig 16). It is bounded below by SB3 (~ 17.62 Ma; HO of *Catapsydrax dissimilis*^103^) and above by SB4 (~ 16.9 Ma; Mid-Rudeis unconformity^54^), spanning ~ 0.72 Ma. Seismic profiles show sub-parallel reflectors with limited continuity (Fig. 17). No lowstand systems tract is preserved. SQ3 includes transgressive (TST3), maximum flooding (MFS3), and highstand (HST3) systems tracts; in ARM-7 and SIDRI-9, only HST3 is preserved due to fossil scarcity.

###### Transgressive systems tract (TST3)

TST3 ranges from 40 m (SIDRI-20) to 190 m (ARS-6), comprising shale and limestone with high gamma-ray values (Figs. 11–12). In ARS-6 and SIDRI-20, Biofacies 8 (*Bolivina–Uvigerina*) and 10 (*Uvigerina–Cassidulina*) dominate, with *Bulimina* sp. (up to 50%) and *Uvigerina* spp. (20–36%), indicating outer shelf to upper slope settings (> 150 m; Sections "Biofacies 8 (Bolvina–Uvigerina biofacies)", "Biofacies 10 (Uvigerina–Cassidulina biofacies)"). P/B ratios (58–83) and TFN (30 individuals/g) reflect continued deepening during transgression.

###### Maximum flooding surface (MFS3)

MFS3 is identified at ~ 16.9 Ma within the upper TST3, marked by peak gamma-ray values and the highest diversity of outer shelf to upper slope biofacies (Biofacies 8 and 10) in ARS-6 and SIDRI-20 (Figs. 11–12), indicating the maximum marine transgression.

###### Highstand systems tract (HST3)

HST3 ranges from 155 m (ARM-7) to 660 m (SIDRI-9), with shale, limestone, sandstone, and siltstone and an aggradational gamma-ray signature (Fig 9, Fig 10, Fig 14, Fig 15, Fig 16). In ARS-6, Biofacies 6 (*Haplophragmoides*), 7 (*Eponides*), and 9 (*Bolivina–Cibicides*) are observed, with *Haplophragmoides* sp. (< 50 m), *Eponides repandus* (0–100 m), and *Cibicides ellisi* (0–50 m; Sections "Biofacies 6 (Haplophragmides biofacies)"–"Biofacies 9 (Bolivina–Cibicides biofacies)"), suggesting inner to middle shelf settings (0–100 m). In SIDRI-20, Biofacies 6 dominates with barren intervals. P/B ratios (10–75) and TFN (10–20 individuals/g) support restricted environments. Barren intervals in ARM-7 and SIDRI-9 are interpreted via aggradational gamma-ray stacking. The water depth shift from TST3’s > 150 m to HST3’s 0–100 m is supported by biostratigraphic evidence (Section "Benthic foraminiferal biofacies"), resolving the reviewer’s concern about Biofacies 6, 7, and 9 in deeper settings.

##### Sequence SQ4

SQ4 corresponds to the Rahmi Member of the Kareem Formation, assigned to the *Praeorbulina glomerosa*Zone (~ 15.99 Ma^130^;) in ARM-7, SIDRI-20, and SIDRI-9 (Figs. 10, 14–16). It ranges from 55 to 170 m thick and comprises shales, sandstones, anhydrite, and limestone deposited in lagoonal to shallow-marine settings. Seismic profiles show sub-parallel reflections (Fig. 17). The sequence is bounded by SB4 (~ 15.99 Ma), and only a lowstand systems tract (LST4) is recognized due to data limitations.

###### Lowstand systems tract (LST4)

LST4 contains Biofacies 3 (*Cibicides–Epistominella*) in SIDRI-20, with *Cibicides* spp. comprising 25–85%, indicating lagoonal conditions (0–5 m; Section "Biofacies 3 (Cibicides–Epistominella biofacies)"). Low P/B ratios (10–15) and TFN (10 individuals/g) reflect regression and restricted marine influence. Blocky gamma-ray trends (Figs. 10, 14–16) support subaerial exposure at SB4.

### Tectonic vs. relative sea-level controls on sedimentation pattern

**Fig. 18:**
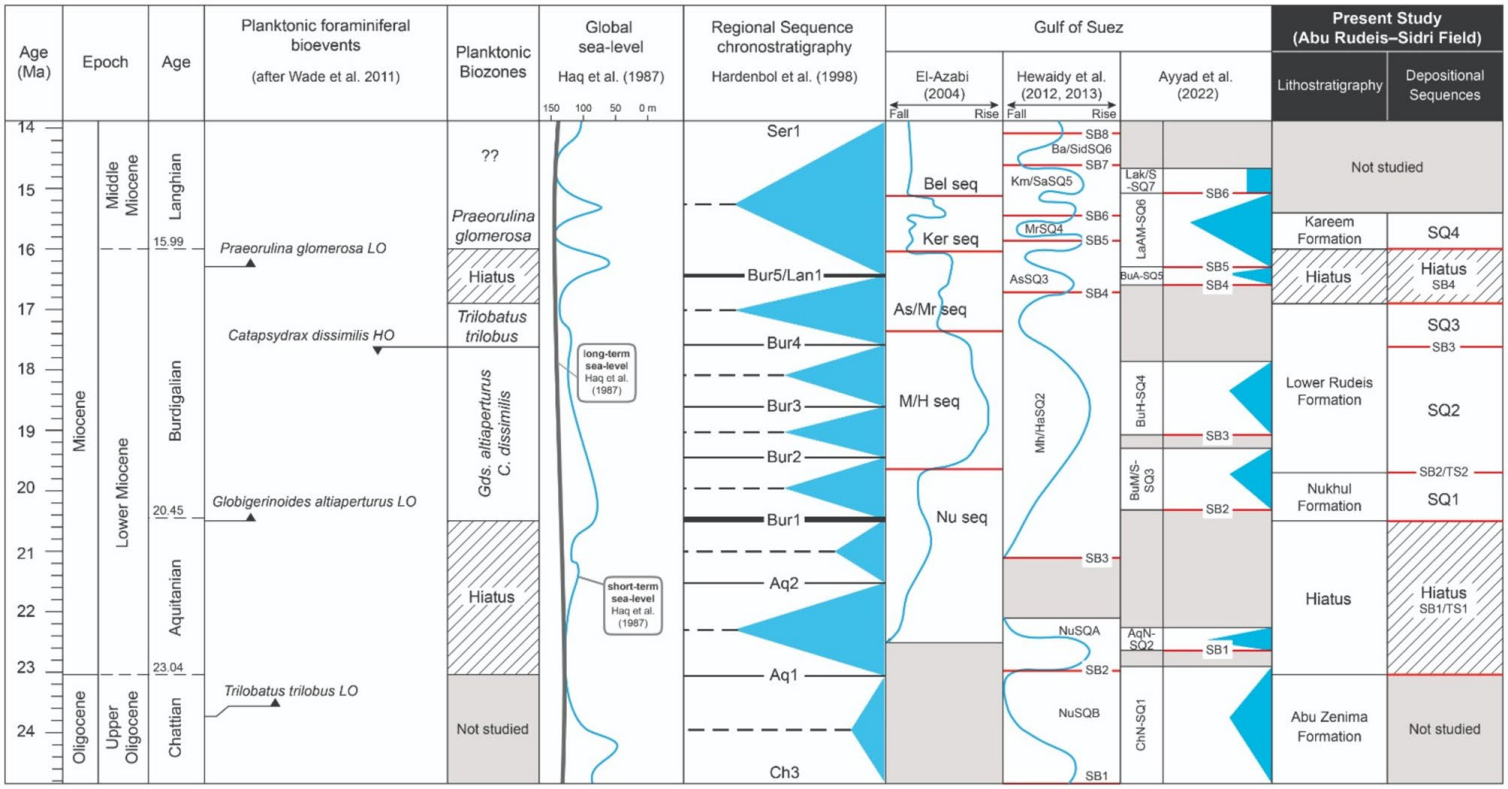
Correlation of the Abu Rudeis–Sidri sequence stratigraphy with local Gulf of Suez studies, the regional sequence framework of^131^, and the global eustatic sea-level curve of^132^, with ages based on the Geological Time Scale 2012^134^.

The Miocene syn-rift stratigraphic architecture of the Abu Rudeis–Sidri Field reflects a dynamic interplay between tectonic subsidence and relative sea-level fluctuations. Based on integrated biostratigraphy, gamma-ray log motifs, and seismic profiles, four third-order depositional sequences (SQ1–SQ4) are recognized. While correlations with regional^131^ and global^132^ sea-level cycles are tentatively proposed, the match is partial and complicated by tectonic overprinting and biochronologic uncertainties (Fig. 18).

The lowermost sequence, SQ1 (early Burdigalian, Nukhul Formation), represents the earliest marine transgression following rift initiation. It unconformably overlies nonmarine Abu Zenima strata across a pronounced composite surface (SB1/TS1), interpreted as a tectonically induced hiatus coinciding with the absence of key Aquitanian planktonic markers such as *Globigerinoides primordius* (Fig 5, Fig 6, Fig 7, Fig 8, Fig 11, Fig 12). This hiatus likely exceeds 2.5 Ma, encompassing much of the Aquitanian. SQ1 thickens basinward (ARM-7, SIDRI-9; Fig. 16), suggesting syn-depositional fault-controlled accommodation. Its transgressive–regressive architecture correlates tentatively with Bur1 of^131^, though faunal constraints are limited.

Sequence SQ2, overlying SB2 (~ 19.7 Ma), corresponds to the early Burdigalian rift climax, marked by deepening to outer shelf–upper slope conditions (Biofacies 1 and 4) and aggradational stacking (Fig. 12). This boundary aligns with the T10 surface of^54^ and approximates the Bur2 eustatic cycle. A similar bounding unconformity has been identified across the Gulf of Suez in the Nukhul-Sudr and Nukhul-Feiran areas^27^^**;**^^41^. The match is plausible given coeval regional tectonism and abrupt deepening observed in both benthic foraminiferal assemblages and gamma-ray patterns.

SQ3 is bounded below by SB3 (~ 17.62 Ma), associated with the HO of *Catapsydrax dissimilis*^103^, and above by the Mid-Rudeis unconformity (SB4; ~ 16.9 Ma^54^;). The sequence shows thickening toward ARS-6 and SIDRI-9 and records a retrogradational–aggradational stacking pattern (Figs. 14–16), culminating in MFS3—a peak flooding surface recognized by high gamma-ray readings and outer shelf to upper slope biofacies (Biofacies 8, 10). While this pattern may align with the Bur3 cycle of^131^, the uncertainty in upper Burdigalian biostratigraphy in these wells tempers confidence in correlation.

Sequence SQ4, initiated at SB4 (base Kareem Formation), reflects a sharp shallowing from marine to restricted-lagoonal settings, as evidenced by the disappearance of planktonic foraminifera and dominance of Biofacies 3 (e.g., *Cibicides*, *Epistominella*). The barren anhydrites of the Rahmi Member (Markha Anhydrite) signal reduced marine connectivity and a major regressive event. This sequence likely corresponds to the Bur5/Lan1 boundary of^131^, although sedimentation across the Burdigalian–Langhian transition is highly variable across the Gulf of Suez^27^^**;**^^41^. Some areas preserve continuous deposition (e.g., Wadi Baba), while others—like the study area—record erosion and hiatus, consistent with uplifted footwall blocks during rift reactivation.

Overall, while approximate correlations with eustatic boundaries^132^^**;**^^131^are proposed, the Gulf of Suez cyclicity is primarily governed by tectonic deformation and block rotation. Half-graben geometries control the distribution of depocenters and associated facies stacking (cf^42^.). The documented unconformities (SB1–SB4) reflect episodic rift activity and uplift rather than purely global sea-level changes. Therefore^133^,global sequences provide a useful reference framework, but must be adapted cautiously in rift contexts, where tectonic processes dominate and age control is hampered by faunal provincialism and biostratigraphic gaps.

Accordingly, our results indicate that tectonic subsidence was the primary control on sequence architecture and accommodation creation. Eustatic influences are discernible at the maximum flooding surfaces (MFS1–MFS3); however, fault-block rotation and tectonic tilting predominantly governed the stratigraphic record. This interpretation aligns with syn-rift models from the Red Sea and East African Rift, yet contrasts with the more eustatically controlled rift margins of Brazil and the North Sea. Sedimentation rate estimates derived from thickness and age data suggest high rates of deposition during SQ2–SQ3 (rift peak), characterized by basinward thickening and localized subsidence above active fault blocks.

## Conclusions

This study establishes a high-resolution chronostratigraphic and sequence stratigraphic framework for the early syn-rift Miocene succession in the Abu Rudeis–Sidri Field, east-central Gulf of Suez, through the integration of foraminiferal biostratigraphy, benthic biofacies, wireline logs, and seismic data from four wells. The studied interval spans the early Burdigalian to early Langhian and is subdivided into four third-order depositional sequences (SQ1–SQ4), bounded by regionally significant sequence boundaries and marked by distinct changes in lithology, gamma-ray response, and foraminiferal assemblages.

Planktonic foraminiferal zonations — including the *Globigerinoides altiaperturus–Catapsydrax dissimilis* Subzone, *Trilobatus trilobus* Zone, and *Praeorbulina glomerosa* Zone, constrain the ages of key sequence boundaries, placing SB1 at the base of the Burdigalian (~ 19.3 Ma), SB3 at ~ 17.62 Ma (HO of *Catapsydrax dissimilis*), and SB4 at ~ 16.9 Ma (Mid-Rudeis Unconformity). Benthic foraminiferal biofacies document paleobathymetric shifts from upper slope (> 150 m) environments during TST2 and TST3, to middle and inner shelf settings (50–100 m) during highstand phases, and ultimately to restricted lagoonal (< 50 m) conditions in SQ4. These facies define transgressive and highstand systems tracts and mark three maximum flooding surfaces (MFS1–MFS3), each associated with peak gamma-ray values and diverse outer shelf–upper slope assemblages.

Accommodation space creation was primarily governed by rift-related faulting and differential subsidence, as evidenced by rapid thickness variations and onlap geometries in seismic profiles. However, eustatic controls are locally expressed at maximum flooding surfaces and during regional transgressive phases. The combined influence of tectonics and eustasy complicates direct correlation with global sea-level curves but enables refinement of the syn-rift depositional history in this sector of the Suez Rift. These results have implications for stratigraphic prediction and reservoir distribution in similar rift-margin settings.

This integrated framework refines the syn-rift stratigraphic model of the Gulf of Suez, illustrating the interplay between tectonic and eustatic controls and offering predictive insights for hydrocarbon exploration, reservoir quality modeling, and gross depositional environment (GDE) mapping. Beyond hydrocarbon implications, the results contribute to general rift-basin models by elucidating how diachronous rift initiation and fault-block tilting influence sequence architecture.

## Supplementary Information

Supplementary Information.

## Data Availability

The datasets used during the current study are not publicly available due to confidentiality agreements but may be made available from the corresponding author, Prof. Mohammad A. Sarhan "the corresponding author, [msarhan@du.edu.eg](mailto:msarhan@du.edu.eg) " upon reasonable request and with permission from the data provider.
