# Supplementary material for: Sequence stratigraphy of the syn-rift miocene succession in the Abu Rudeis-Sidri Field, Gulf of Suez, Egypt
Source: Sci Rep. 2025 Nov 29;15:42906. doi: 10.1038/s41598-025-26923-z (PMC12672564; doi:10.1038/s41598-025-26923-z)
Supplement: Supplementary file 1 — Supplementary Information. [file 41598_2025_26923_MOESM1_ESM.docx]

**Appendix**

**Species systematic list:**

**Phylum: Protista**

**Subphylum: Sarcodina Schmarda, 1871**

**Class: Rhizopoda von Siebold, 1845**

**Order: Foraminiferida Eichwald, 1830**

**Suborder: Textulariina Delage and Hérouard, 1896**

**Superfamily: Astrorhizacea Brady, 1881**

| **Family** | **Subfamily** | **Genus** | **Species** |
| --- | --- | --- | --- |
| **Bathysiphonidae Avnimelech, 1952** | **Bathysiphoninae Avnimelech, 1952** | ***Bathysiphon* Sars, 1872** | ***Bathysiphon taurinensis* (Sacco, 1893)** |
| **Lituolidae Blainville, 1827** | **Ammomarginulininae Podobina, 1978** | [***Ammobaculites***](https://www.marinespecies.org/aphia.php?p=taxdetails&id=112345)**Cushman, 1910** | ***Ammobaculites* sp.** |
| **Haplophragmoididae Maync, 1950** |  | ***Haplophrogmoides* Cushman, 1910** | ***Haplophragmides* sp.** |
|  | **Cyclammininae Marie, 1941** | ***Cyclammina* Brady, 1879** | ***Cyclammina incisa* (Stache, 1864)** |

**Suborder: Miliolina Delage and Hérouard, 1896**

**Superfamily: Miliolacea Ehrenberg, 1839**

| **Family** | **Subfamily** | **Genus** | **Species** |
| --- | --- | --- | --- |
| **Spiroloculinidae Weisner, 1920** |  | ***Spiroloculina* d’ Orbigny, 1826** | ***Spiroloculina communis*** **(Cushman and Todd, 1944)** |
|  |  |  | ***Spiroloculina tenuis*** (**Cžjžek, 1848)** |
| **Hauerinidae Schwager, 1876** | **Hauerininae Schwager, 1876** | ***Quinquloculina* d’ Orbigny, 1826** | ***Quinquloculina seminula* (Linnaeus, 1758)** |

**Suborder: Lagenina Delage and Hérouard, 1896**

**Superfamily: Nodosariaacea Ehrenberg, 1838**

| **Family** | **Subfamily** | **Genus** | **Species** |
| --- | --- | --- | --- |
| **Nodosariidae Ehrenberg, 1838** | **Nodosariinae Ehrenberg, 1838** | ***Dentalina* Risso, 1826** | ***Dentalina baggi*** (**Galloway and Wissler, 1927)** |
|  |  | ***Nodosaria* Lamarck, 1812** | ***Nodosaria catenulata* (Brady, 1884)** |
|  |  |  | ***Nodosaria ovicula* (d´Orbigny, 1826)** |
| **Vaginulinidae Reuss 1860** | **Lenticulininae Chapman, Parr and Collins 1934** | ***Lenticulina* Lamarck 1804** | ***Lenticulina budensis* (Hantken, 1875)** |
|  |  |  | ***Lenticulina hughesi*** **(Kleinpell, 1938)** |
|  |  |  | ***Lenticulina smileyi* (Kleinpell, 1938)** |
|  |  |  | ***Lenticulina luiciana* (Kleinpell, 1938)** |
|  |  |  | ***Lenticulina* sp.** |
| **Lagenidae Reuss, 1862** |  | ***Lagena* Walker, Jacob and Kanmacher, 1798** | ***Lagena apiopleura*** **(Loeblich and Tappan, 1953)** |

**Suborder: Rotaliina Delage and Hérouard, 1896**

**Superfamily: Bolivinacea Glaessner, 1937**

| **Family** | **Subfamily** | **Genus** | **Species** |
| --- | --- | --- | --- |
| **Bolivinidae Glaessner, 1937** |  | ***Bolivina* d’ Orbigny, 1839** | ***Bolivina fastigla* (Cushman, 1936)** |
|  |  |  | ***Bolivina superba*** (**Emiliani, 1949)** |
|  |  |  | ***Bolivina pseudospissa* (Kleinpell, 1938)** |
|  |  |  | ***Bolivina brevior* (Cushman, 1925)** |
|  |  |  | ***Bolivina dilatata* (Reuss, 1850)** |
|  |  |  | ***Bolivina conica*** **(Cushman, 1925)** |
|  |  |  | ***Bolivina saidi*** (**Souaya, 1965)** |
| **Loxostomatidae Loeblich and Tappan, 1962** |  | ***Loxostomoides* Reiss, 1957** | ***Loxostomoides digitata*** **(Arnal, 1984)** |

**Superfamily: Cassidulinacea d,Orbigny, 1839**

| **Family** | **Subfamily** | **Genus** | **Species** |
| --- | --- | --- | --- |
| **Cassidulinidae d,Orbigny, 1839** | **Cassidulininae d,Orbigny, 1839** | ***Globocassidulina* Voloshinova, 1960** | ***Globocassidulina monicana*** **(Cushman and Kleinpell, 1934)** |
|  |  | ***Cassidulina* d'Orbigny, 1826** | ***Cassidulina cruysi* (Marks, 1951)** |
| **Bulimindae Jones, 1875** | **Bulimininae Brady, 1881** | ***Bulimina* d'Orbigny, 1826** | ***Bulimina pupoides*** (**d'Orbigny, 1846)** |
|  |  |  | ***Bulimina* sp1*.*** |
|  |  |  | ***Bulimina* sp2.** |
| [**Buliminellidae**](https://www.marinespecies.org/foraminifera/aphia.php?p=taxdetails&id=465819)**Hofker, 1951** |  | [***Buliminella***](https://www.marinespecies.org/foraminifera/aphia.php?p=taxdetails&id=112277) **Cushman, 1911** | ***Buliminella curta*** **(Cushman, 1925)** |
|  |  |  | ***Buliminella subfusiformis*** **(Cushman, 1925)** |
|  |  |  | ***Buliminella elegantissima*** **(d'Orbigny, 1839)** |
| **Uvigerinidae Haeckel, 1894** | **Uvigerininae Haeckel, 1894** | ***Uvigerina* d'Orbigny, 1826** | *Uvigerina subperegrina* (Cushman and Kleinpell, 1934) |
|  |  |  | ***Uvigerina venusta* (Franzenau, 1894)** |
|  |  |  | ***Uvigerina barbatula* (Macfadyen, 1931)** |
|  |  |  | ***Uvigerina senticosa*** **(Cushman, 1927)** |

**Superfamily: Discorbacea Ehrenberg, 1838**

| **Family** | **Subfamily** | **Genus** | **Species** |
| --- | --- | --- | --- |
| **Discorbidae Ehrenberg, 1838** |  | ***Discorbis* Lamarck, 1804** | ***Discorbis obtusua* (d'Orbigny, 1846)** |
| **Bagginidae Cushman, 1927** | **Baggininae Cushman, 1927** | **Baggina Cushman, 1926** | ***Baggina regularis* (d'Orbigny, 1846)** |
|  |  | ***Valvulineria* Cushman, 1926** | ***Valvulineria minuta*** (**Schubert, 1904)** |
|  |  |  | ***Valvulineria* sp*.*** |
| **Eponididae Hofker, 1951** | **Eponidinae Hofker, 1951** | ***Eponides* de Montfort, 1808** | ***Eponoides repandus*** (**Fichtel and Moll, 1798)** |
|  |  |  | ***Eponoides* sp.** |
| **Cibicididae Cushman, 1927** | **Cibicidinae Cushman, 1927** | ***Cibicides* de Montfort, 1808** | ***Cibicides ellisi ellisi* (Souya, 1965)** |
|  |  |  | ***Cibicides dutemplei* (d'Orbigny, 1846)** |
|  |  |  | ***Cibicides* sp.** |
|  |  | ***Cibicidoides* Thalmann, 1939** | ***Cibicidoides praecinctus* (Karrer, 1868)** |
| [**Pseudoparrellidae**](https://www.marinespecies.org/foraminifera/aphia.php?p=taxdetails&id=465843)**Voloshinova, 1952** | [**Pseudoparrellinae**](https://www.marinespecies.org/foraminifera/aphia.php?p=taxdetails&id=721094) **Voloshinova, 1952** | ***Epistominella* Husezima and Maruhasi, 1944** | ***Epistominella smithi*** (**Stewart and Stewart, 1930)** |

**Superfamily: Nonionacea Schultze, 1854**

| **Family** | **Subfamily** | **Genus** | **Species** |
| --- | --- | --- | --- |
| **Nonionidae Schultze, 1854** | **Nonioninae Schultze, 1854** | **Nonionella Cushman, 1926** | ***Nonionella miocenica* (Cushman, 1926)** |
|  |  | ***Nonion* Montfort, 1808** | ***Nonion scapha* (Fichtel and Moll, 1798)** |
|  |  | ***Pseudononion* Asano, 1936** | ***Pseudononion basispinata* (Cushman and Moyer, 1930)** |
|  |  |  |  |

**Superfamily: Chilostomellacea Brady, 1881**

| **Family** | **Subfamily** | **Genus** | **Species** |
| --- | --- | --- | --- |
| **Chilostomellidae Brady, 1881** | **Chilostomellinae Brady, 1881** | ***Chilostomella* Reuss, 1849** | ***chilostomella ovoidea*** (**Reuss, 1850)** |
| **Gavelinellidae Hofker, 1956** | **Gyroidinoidinae Saidova, 1981** | ***Gyroidina* d'Orbigny, 1846** | ***Gyroidina* sp1*.*** |
|  |  |  | ***Gyroidina* sp2.** |

**Planktonic Foraminifera:**

| **Family** | **Subfamily** | **Genus** | **Species** |
| --- | --- | --- | --- |
| [**Catapsydracidae**](https://foraminifera.eu/querydb.php?family=Catapsydracidae&aktion=suche) **Bolli, Loeblich and Tappan 1957** |  | ***Catapsydrax* Bolli, Loeblich and Tappan, 1957** | ***Catapsydrax dissimilis*, (Cushman and Bermudez, 1937)** |
|  |  |  | ***Catapsydrax* sp*.*** |

**Superfamily: Globigerinacea Carpenter, Parker and Jones,**[**1862**](https://www.tandfonline.com/doi/full/10.1080/14772019.2019.1578831)

**Suborder:** [***Globigerinina***](https://en.wikipedia.org/wiki/Globigerinina) **Delage and Hérouard, 1896**

| **Family** | **Subfamily** | **Genus** | **Species** |
| --- | --- | --- | --- |
| **Globigerinidae Carpenter, Parker and Jones, 1862** | **Globigerininae Carpenter, Parker and Jones,**[**1862**](https://www.tandfonline.com/doi/full/10.1080/14772019.2019.1578831) | ***Globigerina* d’ Orbigny, 1826** | ***Globigerina bulloides* (d'Orbigny, 1826)** |
|  |  |  | ***Globigerina falconensis* (Blow, 1959)** |
|  |  |  | ***Globigerina praebulloides* (Blow, 1959)** |
|  |  |  | ***Globigerina juvenilis* (Bolli, 1957)** |
|  |  |  | ***Globigerina quinquiloba* (Natland, 1938)** |
|  |  |  | ***Globigerina* sp.** |
|  |  | ***Globigerinoides* Cushman, 1927** | ***Globigerinoides altiaperturus* (Bolli, 1957)** |
|  |  |  | ***Globigerinoides subquadratus* (**Brönnimann in Todd et al. 1954) |
|  |  |  | ***Globigerinoides sp.*** |
|  |  | ***Trilobatus* Spezzaferri et al. 2015** | ***Trilobatus immaturus* (Leroy, 1939)** |
|  |  |  | ***Trilobatus quadrilobatus* (d'Orbigny, 1846)** |
|  |  |  | ***Trilobatus*** *sacclifer* (Brady, 1877) |
|  |  |  | ***Trilobatus trilobus* (Reuss, 1850)** |
|  |  | ***Globoturborotalita* Hofker, 1976** | ***Globoturborotalia occlusa* (Blow and Banner, 1962)** |
|  |  |  |  |
|  |  |  | ***Globoturborotalia brazieri* (Jenkins, 1966a)** |
|  |  | ***Dentoglobigerina* Blow, 1979** | ***Dentoglobigerina altispira,* (Cushman and Jarvis, 1936)** |
|  |  |  | ***Dentoglobigerina venezuelana,* (Hedberg, 1937)** |
|  |  |  | *Dentoglobigerina* sp*.* |
|  | **Orbulininae Schultze, 1854** | ***Praeorbulina* Olsson, 1964** | ***Praeorbulina glomerosa*, (Blow, 1956)** |
|  |  |  | ***Paraorbulina sp*** |

**Superfamily: Globorotaliacea Cushman, 1927**

| **Family** | **Subfamily** | **Genus** | **Species** |
| --- | --- | --- | --- |
| **Globorotaliidae Cushman, 1927** |  | ***Globorotalia* Cushman, 1927** | ***Globorotalia praescitula* (Blow, 1959)** |
|  |  |  | ***Globorotalia* sp.** |
|  |  | ***Paragloborotalia* Cifelli, 1982** | ***Paragloborotalia siakensis* (Leroy,1939)** |
|  |  |  | ***Paragloborotalia mayeri***, **(Cushman and Ellisor, 1939)** |

[**Superfamily: Globorotalioidea Cushman,1927**](http://taxonomicon.taxonomy.nl/TaxonTree.aspx?src=0&id=4343193)

| **Family** | **Subfamily** | **Genus** | **Species** |
| --- | --- | --- | --- |
| [**Globorotaliidae**](https://foraminifera.eu/querydb.php?family=Globorotaliidae&aktion=suche) **Cushman, 1927** | **-** | ***Globoquadrina* Finlay, 1947** | ***Globoquadrina* sp*.*** |
| **Globigerinidae Carpenter, Parker and Jones, 1862** |  | **Globorotaloides Bolli, 1957** | ***Globorotaloides suteri* (Bolli, 1957)** |
| [**Globigerinitidae**](https://www.marinespecies.org/foraminifera/aphia.php?p=taxdetails&id=1292679)**Bermúdez, 1961** | [**Globigerinitinae**](https://www.marinespecies.org/foraminifera/aphia.php?p=taxdetails&id=721004)**Bermúdez, 1961** | [***Globigerinita***](https://www.marinespecies.org/foraminifera/aphia.php?p=taxdetails&id=112206)**Brönnimann, 1951** | ***Globigerinita uvula* (Ehrenberg, 1861)** |
|  |  |  | ***Globigerinita* sp*.*** |
| **Globigerinidae Carpenter, Parker and Jones, 1862** | [***Globigerininae* Carpenter et al. 1862**](https://www.marinespecies.org/aphia.php?p=taxdetails&id=721000) | ***Globogerinella* Cushman, 1927** | ***Globogerinella obesa* (Bolli, 1957)** |
